# Photoresponsive Polycations Bearing an Arylazopyrazolium Dye

**DOI:** 10.1021/acsomega.6c00099

**Published:** 2026-03-17

**Authors:** René Steinbrecher, Martin Reifarth, Jiayin Yuan, Christine M. Papadakis, Peter Müller-Buschbaum, Andreas Taubert, André Laschewsky

**Affiliations:** † Institute of Chemistry, University of Potsdam, 14476 Potsdam-Golm, Germany; ‡ Fraunhofer Institute for Applied Polymer Research IAP, 14476 Potsdam-Golm, Germany; § Department of Chemistry, 7675 Stockholm University, 10691 Stockholm, Sweden; ∥ TUM School of Natural Sciences, Department of Physics, 84665 Technical University of Munich, 85748 Garching, Germany

## Abstract

A set of cationic photoresponsive monomers is synthesized from a phenylazopyrazol dye by *N*-methylation followed by ion exchange and is converted by free radical polymerization to the corresponding arylazopyrazolium-bearing polycations. By an appropriate choice of the anion, ionic liquid behavior can be implemented in the monomers. Both the monomers and the polymers retain the outstanding spectroscopic properties of their noncharged arylazopyrazole analogs regarding their quantitative reversible *E–Z* (*trans–cis*) photoisomerization by alternating irradiation with ultraviolet (UV) and green light. At this, they maintain a rather long half-life of the metastable *Z*-isomer despite their much more polar character compared to the noncharged chromophore. Furthermore, the polymers show a characteristic solubility behavior in water. While they dissolve only at elevated temperatures, thus showing an upper critical solution temperature, the polymers remain in solution at temperatures as low as 4 °C. Only rapid cooling or freezing and subsequent thawing induce macroscopic phase separation. Characterization by ^1^H NMR spectroscopy and by measurements of the ζ-potential, the ion mobility, the surface tension, and cryogenic scanning electron microscopy (cryo-SEM) suggests that the monomer is a hydrotrope and that the particular solution behavior is related to the self-assembly of the dye moieties in the aqueous environment.

## Graphical abstract

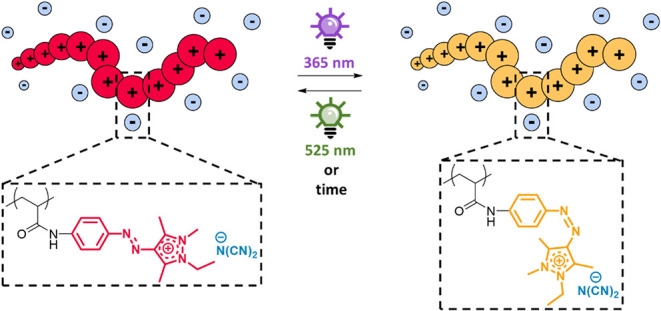

## Introduction

Azo dyes, usually derivatives of azobenzenes, are arguably the most used and studied photoswitches, by virtue of their stability and their reversible *E–Z* (*trans–cis*) photoisomerization, which is virtually free of side reactions.^1–5^ The changes induced may affect, for instance, the optical properties,^6^ the shape,^7^ the orientation,^8^ or the dipole moment and polarity^9^ of the irradiated dyes or of the dye-modified materials, respectively. A relatively new type of azo dye, namely arylazopyrazoles (AAP),^10^ shows several advantages over the traditional azobenzene photoswitch. The absorption maxima of both *E* and *Z* photoisomers are spectroscopically well separated, allowing for a nearly quantitative switch between both isomers with ultraviolet (UV) and green light. Furthermore, AAPs can exhibit extremely long half-lives of the metastable *Z*-isomer, with values up to 1000 days.^10^ For this reason, AAP photoswitches were used in a plethora of applications, including photoresponsive surface functionalizations,^11–15^ host–guest systems,^15,16^ gelators,^17^ rheology control,^18^ controlled drug release,^19^ and enzyme inhibition.^20^ In the course of our investigations on both thermo- and photoresponsive stimuli-sensitive polymers, we found that nonionic polyacrylamide copolymers functionalized with an AAP moiety^21,22^ perform much better with regard to their photoswitchability than the analogues functionalized with classical azobenzene dyes, which have been generally used hitherto.^23–32^ Both types of dye-functionalized copolymers can be adapted to show a lower critical solution temperature (LCST) behavior in aqueous media, as well as to undergo a reversible change of their hydration and solubility upon irradiation by UV or visible light, and the concomitant isomerization from the less polar *E*- to the more polar *Z*-form.^21,32^ Yet in comparison, the polymers functionalized by AAP dyes excel with regard to the nearly quantitative conversion of the *E*- into the Z-state and *vice versa* upon irradiation with near UV light of 365 nm or green light of 525 nm, respectively, as well as by their rather long-lived metastable Z-states (half-life >1 d). Moreover, the cloud points of their aqueous solutions could be shifted significantly more strongly, namely by more than 25 °C upon *E*-to-*Z* photoisomerization, thus broadening the useful temperature window for isothermal photoswitching compared to polymers bearing classical azobenzene dyes.^21,22^ Still, similarly to their polymer analogues functionalized with azobenzene moieties, the AAP-functionalized dually thermo- and photoresponsive stimuli-sensitive polymers suffer from the inherently hydrophobic character of the polymer-bound dyes. Thus, their overall hydrophilicity continuously decreases with increasing dye content. This is accompanied by a concomitant reduction of the cloud point temperature, which eventually falls below 0 °C. This limits the maximum useful content of the photoactive dye in such copolymers to 10–15 mol % even in advantageous cases.^21,32^

In this study, we examine whether the incorporation of AAP dyes containing a charged site can reduce or even remedy the problem of overly hydrophobic dyes. In fact, Gaur and co-workers^33^ successfully quaternized the pyrazole group of a low molar mass AAP chromophore to enhance its water solubility. By systematically varying the substitution pattern on the pyrazolium cation and anion, they studied the structure–property relationship in regards to the absorption spectra, the half-life, the melting points, and the aqueous solubility. Therefore, we design a set of cationic monomer analogs **2a–e** of the previously employed AAP-bearing monomer AAPEAm **1** (Figure 1), in an attempt to modulate the water solubility of the monomers, and also of the polymers formed thereof, by the additional ionic site in the hetero aromatic ring. In particular, we introduce diverse low molar mass counterions, with the aim to vary their relative positions in the empirical Hofmeister series and, thus, to modulate the water solubility of the resulting ionic monomers.^34–36^ The limited studies comparing the anions employed suggest an increasing chaotropic character of the anions in the order of NO_3_^–^ < CF_3_SO_3_^–^ < BF_4_^–^ ∼ I^–^ < N­(CN)_2_^–^, which typically favors the water solubility for proteins and polyzwitterions, while reducing it for synthetic polycations. Further, such anions tend to lower the melting points of organic ammonium salts markedly, thus inducing ionic liquid behavior.

**Figure 1:**
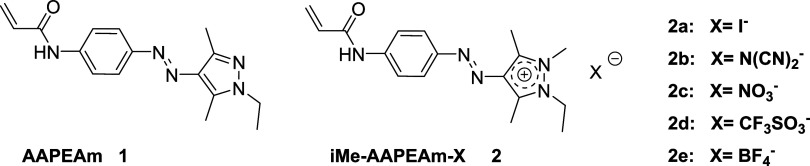
Chemical structures of the nonionic arylazopyrazole (AAP)-functionalized precursor monomer **AAPEAm 1** and of the derived cationic monomers **iMe-AAPEAm-X 2a–e** with counterions **X** ^–^ (iodide, dicyanamide, nitrate, trifluoromethanesulfonate (triflate), and tetrafluoroborate).

In this study, we describe the synthesis, molecular characterization, and free radical polymerization of the AAP-bearing cationic monomers **2a–e**. We study the spectroscopic properties of both the monomers and polymers and explore the aqueous solution behavior exemplarily for the dicyanamide salt **2b** being a water-soluble ionic liquid (IL) and its homopolymer **P-2b** representing an amphiphilic polycation by various methods.

## Experimental Section

### Materials

Silver tetrafluoroborate (99%, Alfa Aesar), silver triflate (97%, Apollo Scientific), silver nitrate (<99.8%, Thermo Fisher Scientific), sodium dicyanamide (96%, Sigma-Aldrich), acetonitrile (<99.5%, VWR, ACN), and methyl iodide (99%, Acros Organics, stabilized) were used as received. The synthesis of the arylazopyrazole-functionalized acrylamide (AAPEAm **1**) was described before.^21^ The preparation of silver dicyanamide starting from sodium dicyanamide followed a literature procedure.^37^

Methanol (MeOH) for polymerization reactions was distilled and stored under a nitrogen. 2,2′-Azobis­(2-methylpropionitrile) (AIBN, <98%, Sigma-Aldrich) was recrystallized from distilled methanol. Water for purification and sample preparation is deionized. Water used for surface tension measurements was first deionized and additionally purified with a Millipore Milli-Q Plus water purification system (Merck) and is in the following called milli-Q water. Aluminum oxide (AlOx, Sigma-Aldrich, activated, basic, Brockmann I, standard grade, ∼150 mesh, 58 Å) was used for the filtration of the monomer to remove traces of the inhibitor.

## Methods

^1^H and ^13^C nuclear magnetic resonance (NMR) spectra were recorded on an Ascend 400 MHz spectrometer (Bruker). The spectra were evaluated with Topspin software, version 4.4. The chemical shifts δ are given in ppm. The residual proton signal of the solvent or, respectively, its C signal was used to calibrate the ^1^H and ^13^C NMR spectra. The ^13^C NMR spectra were recorded in proton decoupled mode.

A Varian 640-IR Fourier transform infrared spectrometer was used for Fourier transform infrared (FT-IR) spectroscopy, and the samples were analyzed with a Specac Goldengate ATR unit. The software Resolutions Pro version 5.1.0.829 was used for data analysis.

Ultraviolet–visible (UV–vis) measurements were made on either a Genesys 150 (Thermo Fisher Scientific) or a dual-beam spectrometer model Cary500 (Agilent). Samples (concentration = 4 mg L^–1^ in water or methanol) were prepared at least 1 d before the measurement and were stored at ambient temperature (about 21 °C) in the dark.

Turbidimetry measurements were performed on a dual-beam spectrometer, Cary500 (Agilent). Samples (concentration = 1 g·L^–1^ in water) for turbidimetry were prepared at least 1 d before the measurement and were annealed at room temperature in the dark. The samples were heated at a rate of 0.5 K min^–1^, and the transmission at 600 nm was recorded at 0.25 K.

Electrospray ionization (ESI) mass spectra were recorded using a Bruker maXis mass spectrometer (Quadrupole, Time of Flight) equipped with an ESI source in positive ion mode.

All UV-light irradiations were carried out with an Alonefire SV47 12 W 365 nm UV flashlight (Shenzhen Shiwang Technology Co. Ltd., Shenzhen/China). The samples were irradiated with an irradiance of 200 mW·cm^–2^ as determined by an optical power meter PM100D with a sensor S170C (Thorlabs, Newton/USA). Blue and green light irradiations were performed with a home-built setup using a high-power light-emitting diode (LED) (3 W) as irradiation source from LEDs-and-more (Berlin/Germany).

Aqueous size exclusion chromatography (SEC) was conducted with the addition of 0.1 M NaCl and 0.3 vol % of formic acid. Measurements were performed at a flow rate of 1 mL·min^–1^ at 40 °C. The stationary phase was a 300 × 8 mm^2^ PSS NOVEMA Max column. Measurements were executed with synchronous UV and RI detection. Samples were filtered through 0.45 μm poly­(tetrafluoroethylene) filters (Carl Roth, Karlsruhe/Germany) with an injected volume of 100 μL. Poly­(vinylpyrrolidone) standards (PSS, Mainz/Germany) were used for calibration.

ζ-Potential, particle size, molar mass, and ion mobility measurements were conducted on a Zetasizer Nano Series (Malvern Panalytical, Malvern/U.K.) at ambient temperature. ζ-Potential was measured with a dip cell. Samples (concentration = 1 g·L^–1^ in water) were prepared at least 1 d before the measurement, stored at room temperature in the dark, and filtered through a syringe filter with the pore size of 0.2 μm immediately before the measurement. Dynamic light scattering (DLS) measurements were performed in the backscattering mode, and the size distribution was weighted by intensity.

Differential scanning calorimetry (DSC) was performed on a DSC 3+ instrument (Mettler Toledo) using aluminum crucibles. Samples were heated from 0 to 250 °C with a heating rate of 10 K·min^–1^ under a nitrogen atmosphere. The DSC data was processed with the STARe software, version 16.4. The onset of the endothermic melting peak was defined as the melting point (*T*_m_).

Cryogenic scanning electron microscopy (cryo-SEM) specimens were prepared by plunge-freezing the polymer solution (concentration = 5 g·L^–1^ in water, filtered through a syringe filter with a pore size of 0.2 μm) into cooled liquid nitrogen. The liquid nitrogen was cooled to −210 °C by filling a dewar vessel in a vacuum chamber and removing the gas phase until the nitrogen froze, then the pressure was slightly increased to thaw the solid nitrogen, and then directly reduced again. Over several pump-and-thaw cycles, solid nitrogen aggregated in the bottom of the vessel, indicating that the aimed temperature was achieved. The frozen droplet was transferred under liquid nitrogen into the EM VCM (Leica) mounting chamber, where the specimen was fixed on a cryo-EM stage and then collected with the EM VCT500 (Leica) shuttle. The final step of the preparation was performed in the EM ACE600 (Leica) preparation chamber, where the surface of the specimen was checked and polished with a blade under cryogenic conditions if needed (temperature < −160 °C, pressure <1·10^–7^ bar). Finally, the specimen was transferred with a shuttle to the scanning electron microscope (SEM) JEOL JSM IT-800 and analyzed. After termination of all measurements, the specimen was stored in the preparation chamber, and the temperature increased to 25 °C, while keeping the pressure low. With this process, the droplet was freeze-dried and could be imaged with noncryogenic EM. Noncryo SEM was performed on a table-top TM3000 (Hitachi). After energy-dispersive X-ray spectroscopy (EDS) measurements, the freeze-dried polymer samples were gold-coated with a JFC-1200 fine coater (Jeol), and images with enhanced contrast were recorded.

The surface tension of aqueous solutions was measured with a Krüss digital tensiometer model K 10 ST (Hamburg/Germany). All glassware used to prepare the monomer and polymer solutions was cleaned with concentrated sulfuric acid, rinsed with milli-Q water, and then dried in an oven or by calcining it with a blowtorch. Samples depicted as “*E*-state” were irradiated with green light (525 nm) for 10 min, while “*Z*-state” samples were irradiated with UV light (365 nm) before measuring the surface tension. All measurements were made in triplicate at 20.0 ± 0.1 °C, and the average with standard deviation as error bar was plotted versus the polymer concentration.

## Results

### Syntheses

Starting from the known arylazopyrazole-modified acrylamide **1** (AAPEAm),^21^ the cationically charged derivatives are obtained in two synthesis steps by *N*-alkylation and subsequent ion exchange, as schemed out exemplarily for monomer **2b** in Scheme 1 (for detailed synthesis procedures, analytical data, ^1^H and ^13^C NMR spectra, and FT-IR spectra, see Supporting Information; Schemes S1–S4, Figures S1–S19).

**Scheme 1:**
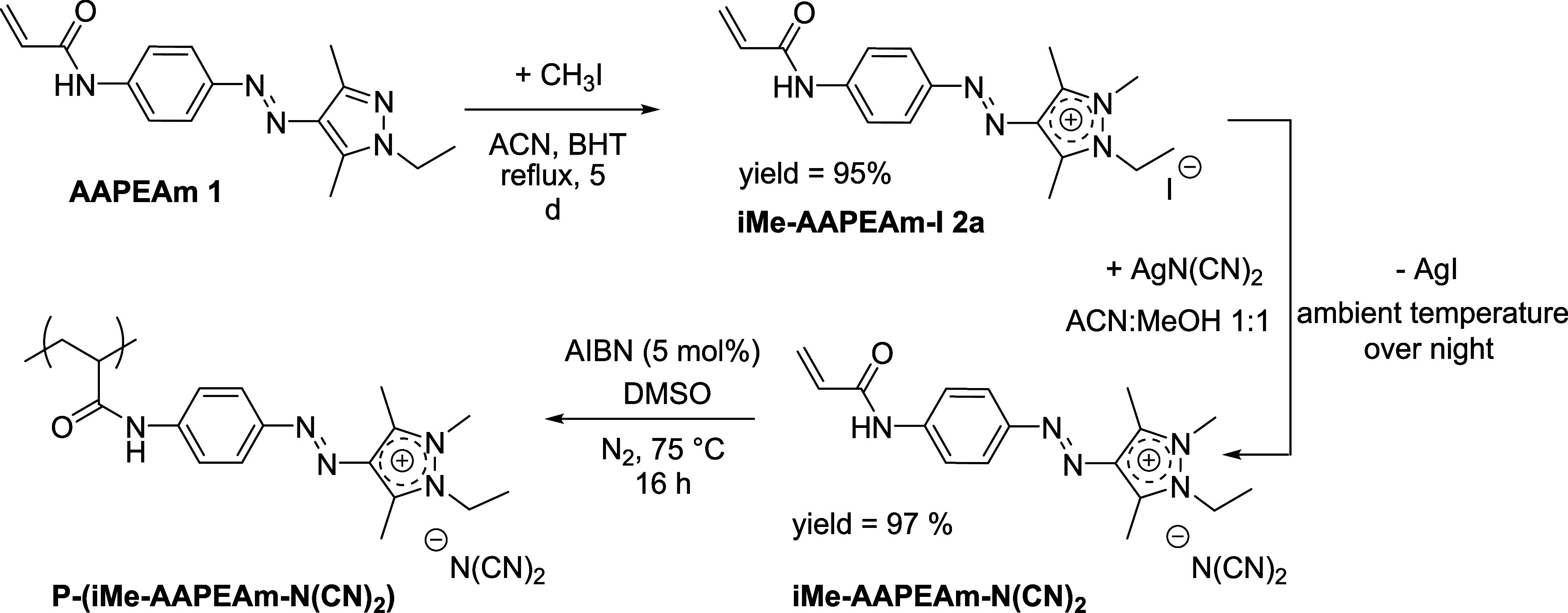
Synthesis of the Monomer iMe-AAPEAm-N­(CN)_2_ **2b** and Its Free Radical Polymerization Using AIBN as the Thermal Initiator

Due to the low reactivity of the pyrazole moiety, a reaction time of 5 d and a large excess of alkylating agent methyl iodide (CH_3_I) are necessary to reach quantitative conversion, in agreement with previous reports on alkylation of pyrazoles.^33^ Small amounts (5–10 mg) of 2,6-di-*tert*-butyl-*p*-cresol (BHT) are added as an inhibitor to scavenge free radicals that might possibly form during the prolonged heating. Explorative quaternization attempts with other alkylating reagents, *i.e*., ethyl, pentyl, or benzyl iodide, yield only very low conversions under the same conditions.

The final monomer, iMe-AAPEAm-N­(CN)_2_, is synthesized via an ion exchange with AgN­(CN)_2_: the precursor iMe-AAPEAm-I is dissolved, the silver salt is suspended in excess, and the product is obtained from the filtrate.^37^ While the exchange is confirmed qualitatively with FT-IR and ^13^C NMR spectroscopies, EDS measurements prove the quantitative removal of iodide (*cf*. EDS spectra in the Supporting Information, Figures S20–S26).

Also, the free radical polymerization of the cationic dye monomers is not self-evident. While the starting material AAPEAm **1** easily reacted in free radical polymerizations,^21^ the cationic derivative only polymerizes when engaging relatively high amounts of the initiator AIBN (5 mol %). The reason for this behavior is unclear yet. As the monomer was carefully purified, as corroborated by the analytical data, it can be ruled out that residual BHT hampers the polymerization. In any case, true polymers are produced, with an apparent number-average molar mass *M*_n_ = 9.2·10^3^ g·mol^–1^ and a dispersity *Đ* = 1.4 (calibration by poly­(ethylene glycol) standards) according to analysis by size exclusion chromatography (SEC).

As described for the dicyanamide salt **2b**, the analogous monomers **2c**–**e** with different counterions (NO_3_^–^, CF_3_SO_3_^–^, BF_4_^–^) are synthesized by ion exchange of the iodide precursor **2a** using the corresponding silver salts and homopolymerized applying the same polymerization procedure. Again, equally high amounts of initiator AIBN (5 mol %) are required for successful polymerization, yielding 83%, 83%, 95%, and 57% of polymers **P-2a**, **P-2c**, **P-2d**, and **P-2e**, respectively. Accordingly, the difficulties achieving sufficiently high monomer conversion are related rather to the phenylazopyrazolium cation, which might act as a retarder, than to the various anions of the dye salts. In contrast to polymer **P-2b**, however, we could not establish an appropriate column system and eluents/conditions for the SEC analysis of the other polycations. This is attributed to their general amphiphilic character of being hydrophobized polyelectrolytes, with the tendency for aggregation and adsorption on both polar and apolar surfaces.

Further details of the syntheses and the corresponding spectra are found in the Supporting Information on S5–S9 and Figures S8–26, respectively.

### Melting Behavior of Arylazopyrazolium Monomers

Figure 2 summarizes the melting points (*T*_m_) of the monomers studied, including the *T*_m_ of the precursor AAPEAm for reference.

**Figure 2:**
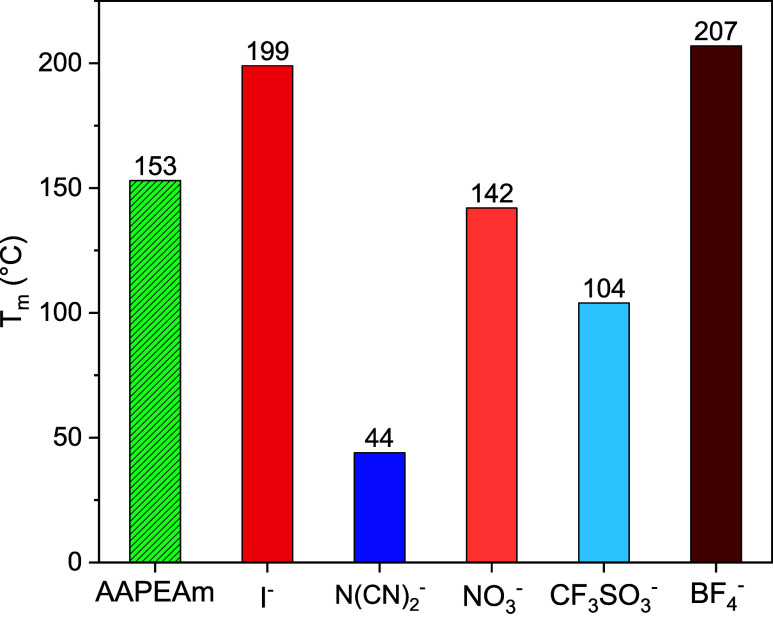
Melting points (*T*_m_) of the cationic monomers **iMe-AAPEAm-X 2a-e** in dependence on the nature of the counterion **X**, as determined by DSC, in comparison to the nonionic precursor **AAPEAm 1**. Numbers on top of the columns show the observed *T*_m_.

The *T*_m_ values span a broad temperature range, being in parts higher, but also in parts lower than *T*_m_ of the precursor. The dicyanamide monomer **2b** (X^–^ = N­(CN)_2_^–^) exhibits the lowest *T*_m_ value of 44 °C, while monomer **2d** with the triflate counterion also shows a relatively low-lying *T*_m_ value of 104 °C. In contrast, the *T*_m_ value of 142 °C of the nitrate salt **2c** is similar to the *T*_m_ of precursor **1**, while the *T*_m_ values of iodide **2a** (199 °C) and tetrafluoroborate **2e** (207 °C) are notably higher. It is interesting to note that for standard ILs, the melting points increase differently, *e.g*., for 1-butyl-3-methylimidazolium salts, the melting points increase in the order BF_4_^–^ ∼ I^–^ < N­(CN)_2_^–^ < CF_3_SO_3_^–^ < NO_3_^–^,^38–42^ while for the slightly different substituted 1-ethyl-3-methylimidazolium salts, the melting points increase in the order N­(CN)_2_^–^ < CF_3_SO_3_^–^ < BF_4_^–^ < NO_3_^–^ < I^–^.^43–47^ This demonstrates that the salt’s behavior cannot be predicted by merely summing up specific increments for the underlying anion and cation.

### Visible Solubility Behavior

Remarkably, the homopolymers of all monomers synthesized in the present work, *i*.*e.*, for all anions, are water-soluble at elevated temperatures and show an upper critical solution temperature (UCST) with clearing point temperatures (*T*_CLP_) for 1–5 g·L^–1^ solutions in the range of 60–75 °C. Surprisingly, cooling slowly to 4 °C does not induce the precipitation of the dissolved polymers, as is normally observed for UCST polymers. Only freezing and thawing or rapid cooling with liquid nitrogen enforce the macroscopic phase separation and induce polymer precipitation, resulting in a suspension that sediments slowly over several hours. As shown in the Supporting Information, we do not observe a decrease in transparency by decreasing the temperature of a once-dissolved polymer solution (*cf*. Figures S27 and S28). This unusual behavior suggests a kinetically trapped system of the polyelectrolyte when dissolved in water. This is possibly based on a self-assembly of the polymers mediated by the chromophore or microphase separation, a phenomenon that has already been occasionally reported for other polymerized ionic liquids.^48–50^

Since all polymers show similar solubility behavior, further investigations were exemplarily performed with the homopolymer **iMe-AAPEAm-N­(CN)**_**2**_, **2b**, which can be classified as a polymerized ionic liquid.^51–54^

### Molecular Solubility Behavior

The solution state of the polymers can be qualitatively assessed with liquid-state ^1^H NMR spectroscopy, as only fully dissolved and freely moving polymer chains produce a full intensity signal, while partially aggregated chains show considerably broadened, attenuated, or even no signals at all. Therefore, **P-2b** is dissolved in three perdeuterated solvents of differing polarity and H-bonding capability, namely dimethylsulfoxide (DMSO–D_6_), methanol (MeOD-D_4_), and water (D_2_O). The corresponding ^1^H NMR spectra are shown in Figure 3.

**Figure 3:**
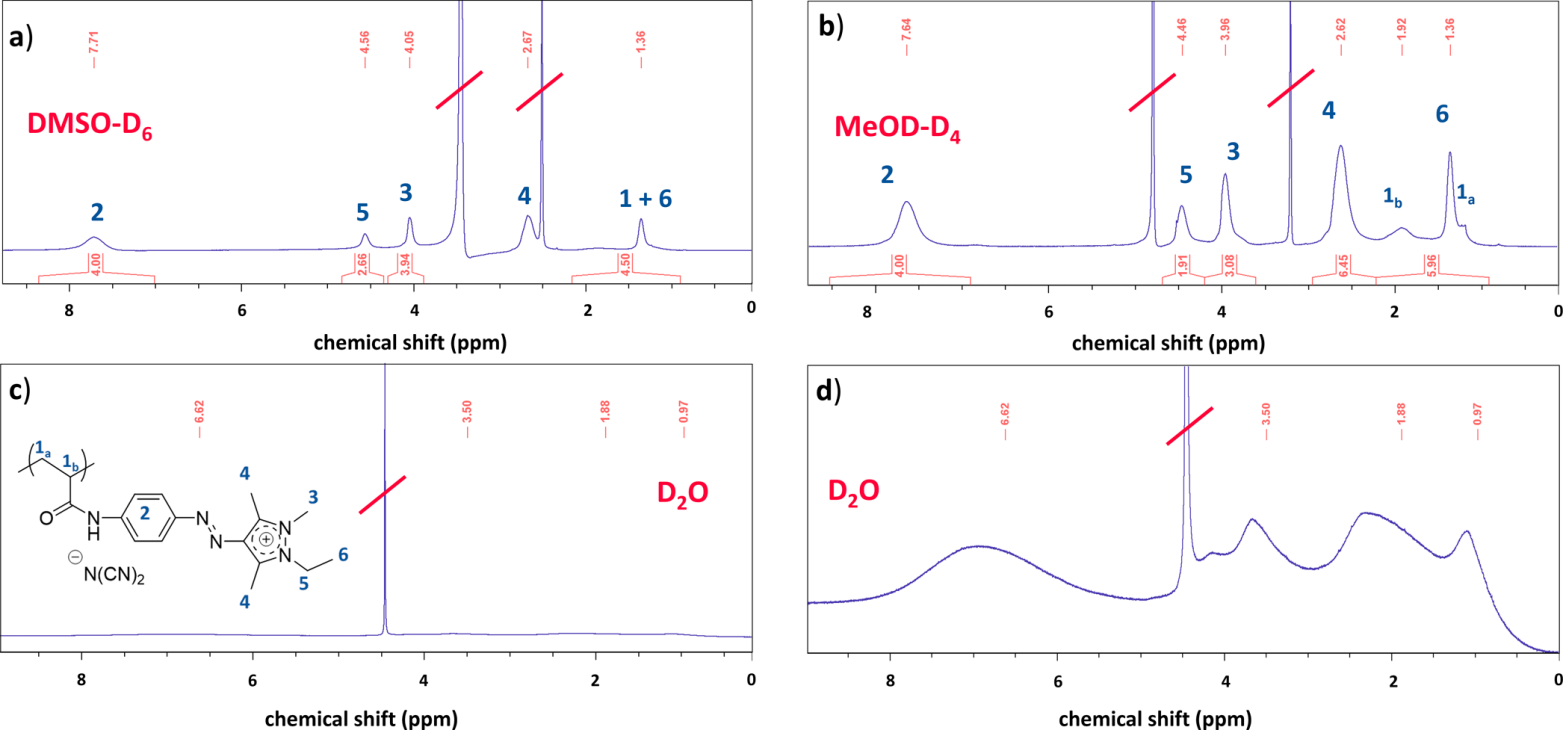
Liquid-state ^1^H NMR of **P-(iMe-AAPEAm-N­(CN)**_**2**_ **)** (**P-2b**) in different solvents (concentration of 24 g·L^–1^): Observed signals in the spectra with deuterated DMSO–D_6_ (a) or methanol MeOD-D_4_ (b) are assigned to the corresponding proton via blue numbers; (c, d) spectrum in D_2_O, with the signal intensities strongly magnified in part (d). All measurements were conducted at ambient temperature, and the residual proton and water peaks are marked with a red diagonal slash.

^1^H NMR spectra obtained in DMSO–D_6_ and MeOD-D_4_ show distinct polymer signals, while the spectrum in D_2_O shows broad and attenuated or even missing signals at the expected chemical shifts. This finding suggests that the polymers are fully dissolved in MeOD-D_4_, but that their mobility is strongly restrained in D_2_O at ambient temperature, although remaining visually clear. This might indicate that the polymers self-assemble in water, thereby reducing the contact of their hydrophobic parts with the water molecules.

### Spectroscopic Properties of the Photoswitch

The UV–vis spectra of the monomer **iMe-AAPEAm-N­(CN)**_**2**_ in methanol, which is a common solvent for these cationic azo dyes and their uncharged precursor **AAPEAm 1**, differ only slightly from that of AAPEAm **1** in methanol (cf. Supporting Information, Figures S29 and S30).^21^ All of these phenylazopyrazoles show a main π–π* absorbance band in the near UV range around 350 nm and an incompletely resolved weaker *n*–π* band in the visible range around 430 nm. In detail, the maximum of the π–π* band of 354 nm for **1** is slightly blue-shifted to 353 nm after quaternization of the pyrazole moiety, while the *n*–π* bands of **1** are shifted from 447 to 438 nm for monomer **2b**. Also, the phenylazopyrazolium dye shows only weak solvatochromism (*cf*. Figure 5). The small differences found for the phenylazopyrazoles differ strongly from the marked red shifts observed in the spectra of phenylazopyridines after quaternization and from the marked solvatochromism and halochromism,^55–57^ which is attributed to the thereby markedly increased push–pull substitution pattern of such heterocyclic azo dye.

The favorable UV–vis spectroscopic properties of the arylazopyrazole chromophores arise from the distinct absorption maxima of the *E-* and *Z*-isomers, allowing for the selective excitation of each photoisomer and thus enabling near-quantitative switching between the two states.^10,21^ Figure 4a presents the superimposed UV–vis spectra of all monomers before and after irradiation by UV light in water. Only small differences are observed for the various salts; *i.e*., the loosely associated counterions only have a minimal influence on the electronic configuration of the azo pyrazolium group, as might be expected. Therefore, the photoswitching behavior was studied exemplarily for the dicyanamide salt **2b**, as shown in Figure 4b. In the photoswitching experiment, the sample was first measured in the dark and then irradiated alternately with UV light (I–III) and deep blue (450 nm), cyan (480 nm), and deep green (525 nm) visible light.

**Figure 4:**
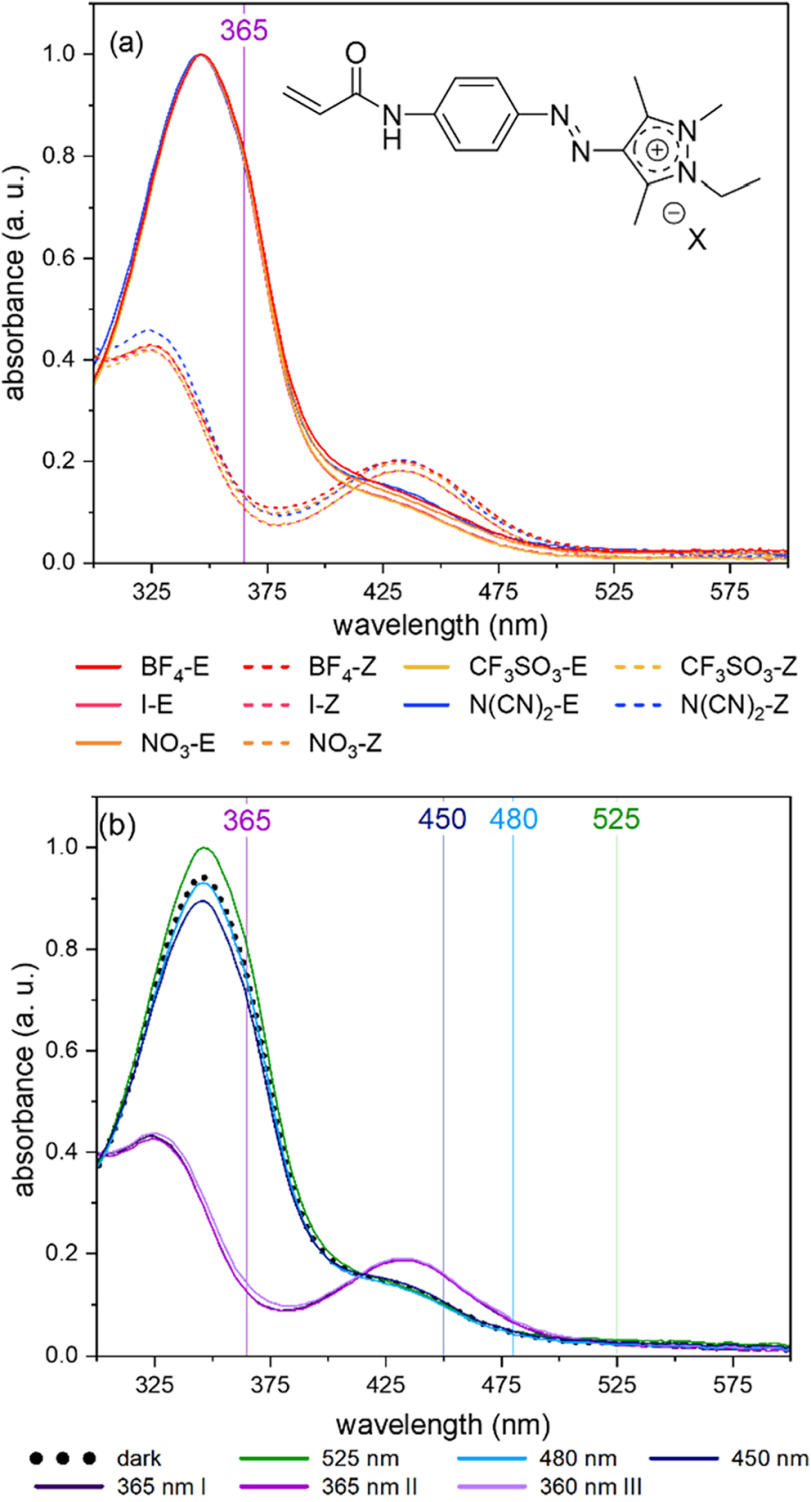
(a) UV–vis spectra of the arylazopyrazolium monomers **2a**–**e** before (continuous lines) and after (dotted lines) irradiation in water, corresponding to the *E-* and *Z*-states; (b) normalized absorption spectra of **iMe-AAPEAm-N­(CN)**_**2**_ **(2b**) before irradiation (“dark”) and after alternating irradiation with UV light (purple lines I–III) and visible light of various wavelengths in water. “*E*” in the labels refers to the E*-*state, and “Z” refers to the *Z*-state (all polymer concentrations 0.004 g·L^–1^, ambient temperature). The absorbance is given in arbitrary units.

The photocycling behavior of the chromophore demonstrates that it is possible to photoisomerize the majority of the isomers from the *E-* to the *Z*-state, and *vice versa*, using light (*cf*. Figure 4b). Irradiation with blue light (450 and 480 nm) induces the return to the *E* form to a large extent, but does not fully restore the initial nearly quantitative *E* content of the dark state (*i*.*e.*, the sample before any irradiation). In contrast, green light (525 nm) restores completely and even surpasses the initial *E–Z* ratio due to a small amount of *Z*-isomer (around 1%) present already in the starting (dark) sample (*cf*. Figures S31–S33). Overall, we find that the introduction of a charge on the heterocycle of the arylazopyrazole does not impair the chromophore’s spectral and photoswitching properties, as the behavior of the precursor arylazopyrazole dye **AAPEAm 1** and the salt **iMe-AAPEAm-N­(CN)**_**2**_ **2b** derived by methylation are very similar.

Next, we address the extent to which the polymerization of the cationic dye monomer affects the absorption properties and assess whether the solvent influences the absorption behavior of the polyelectrolyte formed. As shown in Figure 5a, polymerization neither significantly affects the absorption spectra of the dye nor the ability to selectively excite either the *E*-state with UV light or the *Z*-state with green light.

**Figure 5:**
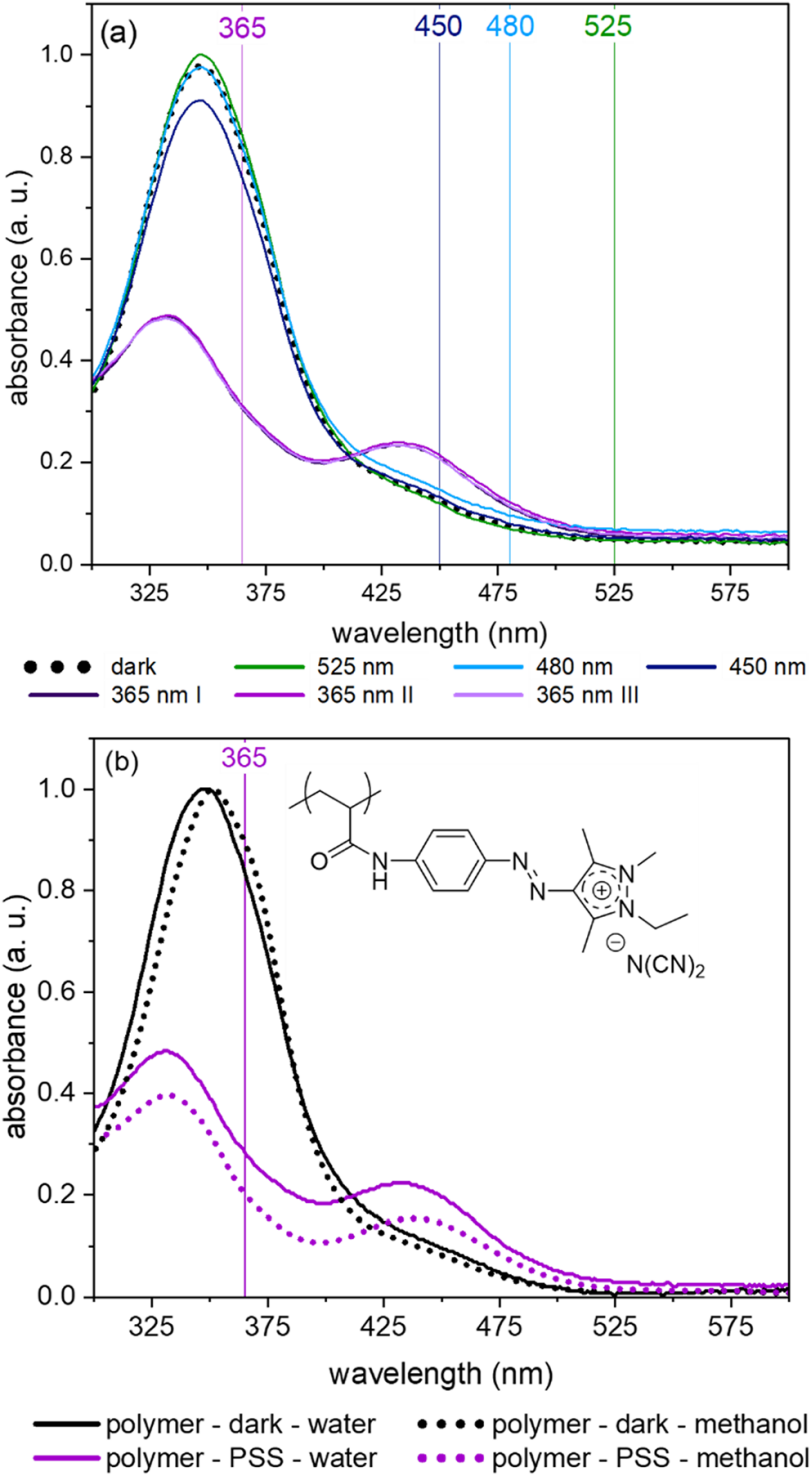
(a) Normalized absorption spectra of **P-(iMe-AAPEAm-N­(CN)**_**2**_ **) P-2b** in water before irradiation (“dark”) and after alternating irradiation with UV light (I–III) and visible light of different wavelengths; (b) comparison of the spectra of **P-2b** in water (continuous lines) and methanol (dotted lines) before (“dark,” in black) and after irradiation (in purple) with 365 nm UV light (concentration = 0.004 g·L^–1^, ambient temperature).

The comparison of the homopolymer’s absorption spectra in water and in methanol exemplifies that the solvent has only a small solvatochromic effect on the chromophore’s properties, causing a slight red shift of the π→π* band for the *E*-isomer by about 2 nm in methanol while retaining the general spectral profile (Figure 5b). The absorption spectrum of the Z-state appears to be unaffected by variation of the solvent. Accordingly, the microphase separation suggested by the ^1^H NMR experiments (see Figure 3) does not restrict the chromophore’s freedom of motion in water to an extent that it interferes with an effective photoisomerization of the azo dye.

The lifetime of the *Z*-isomer formed in the photostationary state (PSS) of **P-(iMe-AAPEAm-N­(CN)**_**2**_ **) P-2b** in water is assessed as well. As shown in Figure 6, the spectrum of the aqueous sample, which was initially measured in the dark, is used as a reference for the base *E*-state. After the sample was irradiated at room temperature with 365 nm UV light for 5 min, it was measured immediately to assess the spectrum in the PSS, and subsequent spectra were recorded at fixed intervals. Complementary ^1^H NMR spectra of the chromophore before and after UV irradiation show that irradiation converts an almost pure *E*-isomer-containing sample into one containing almost exclusively the *Z*-isomer (*cf*. Figures S31–S33). The recovery from the PSS to the dark state is indicated by a line, with the first half of the relaxation being highlighted in red color, ending at the halfway point and indicating an estimated half-life of around 7 h.

**Figure 6:**
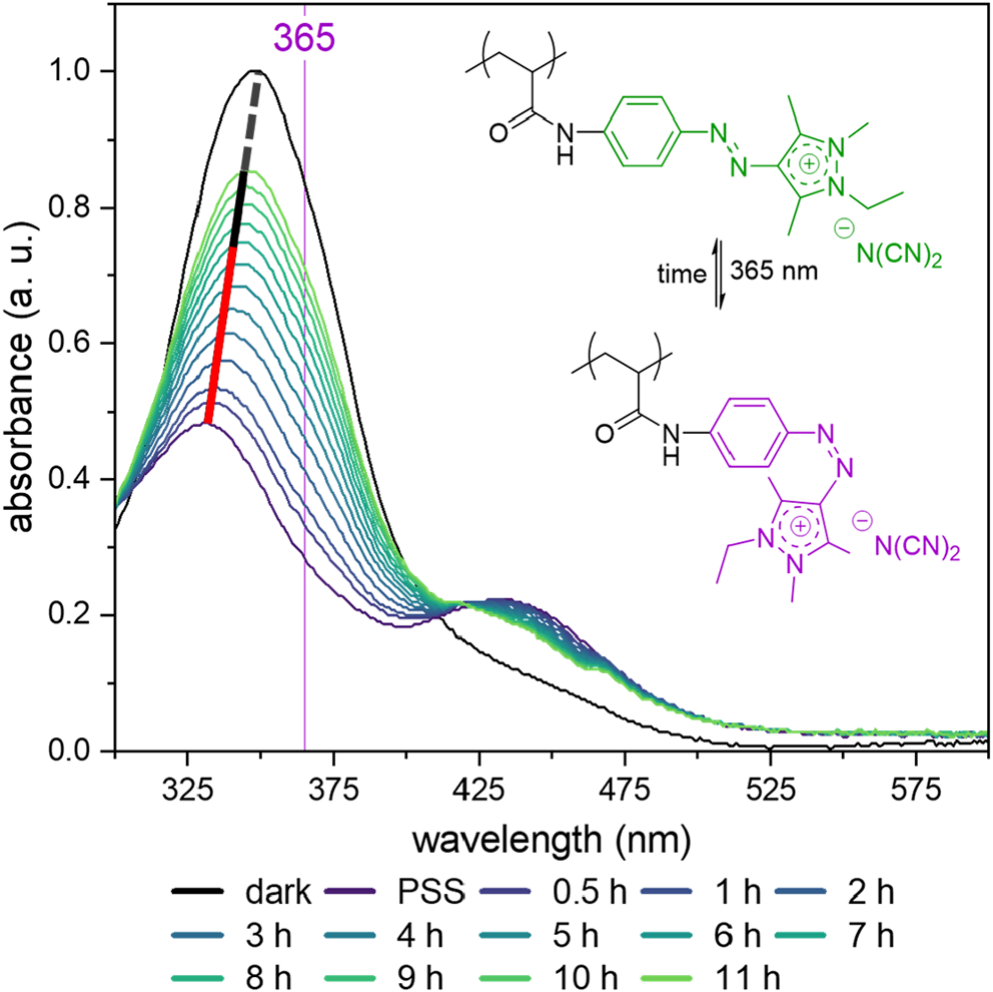
UV–vis spectra showing the relaxation of the photostationary state of **P-(iMe-AAPEAm-N­(CN)**_**2**_ **) P-2b** upon UV irradiation in water (concentration = 0.004 g·L^–1^) over time at ambient temperature. The thin purple line indicates the position of the irradiation wavelength. The sample was measured before irradiation (dark), immediately after 5 min of UV-light irradiation (PSS), and then at fixed intervals. The red-and-black line is meant as a guide to the eye for the ongoing evolution from the PSS back to the dark state, and the red part representing the first half of the relaxation and ending at the halfway point, marking the estimated half-life.

The *Z*-isomer present in PSS exhibits a half-life of approximately 7 h at room temperature. This value is relatively short for a neutral arylazopyrazole dye in solution,^10,21^ yet still sufficiently long for experiments requiring a single irradiation rather than continuous irradiation throughout the experiment. The analogous experiment is also conducted for monomer **2b** in aqueous solution (*cf*. Figure S34), revealing an even shorter half-life of about 2.5 h.

The uncharged precursor dye monomer AAPEAm showed half-lives for the thermal relaxation from the *Z*- to the *E*-isomer of 29 h.^21^ Thus, one might tentatively attribute the markedly reduced half-lives of **2b** and **P-2b** to the increased push–pull character of the chromophore’s substitution pattern due to the methylation. This behavior is well documented for classical azobenzene dyes that have a push–pull substitution pattern,^58^ *e.g*., having an electron-donating group such as an amide and an electron-withdrawing group such as the ammonium motif on the opposite ends of the π-conjugated aryl-diazo moiety, and also for 4-arylazopyridines and their *N*-methylated cationic analogs.^58^ However, a markedly increased push–pull character of azo dyes is typically accompanied by a strong bathochromic shift of the absorbance maximum, which is not observed for **AAPEAm** and its **iMe-AAPEAM-X** analogs. Moreover, the half-life of monomer **2b** amounts to about 224 h in methanol, as measured by ^1^H NMR (*cf*. Figure S35), while for polymer **P-2b**, the half-life is about 96 h (*cf*. Figure S36). The half-lives in methanol solution correspond roughly to the ones of the uncharged AAPEAM-based analogs.^21^ Accordingly, the strong reduction of the half-lives in water seems to be related to the solvent properties. Presumably, the self-assembly of the ionic azo dye in water as suggested by the ^1^H NMR spectra (*cf*. Figure 3) accelerates the *Z*-to-*E* relaxation process strongly.

### Self-Assembly in Aqueous Solution

The behavior of polymer **P-2b** in aqueous solution upon photoisomerization is investigated in more detail. ζ-Potential and ion mobility are measured using laser Doppler microelectrophoresis, revealing at best small changes upon irradiation by UV light (Figure 7a).

**Figure 7:**
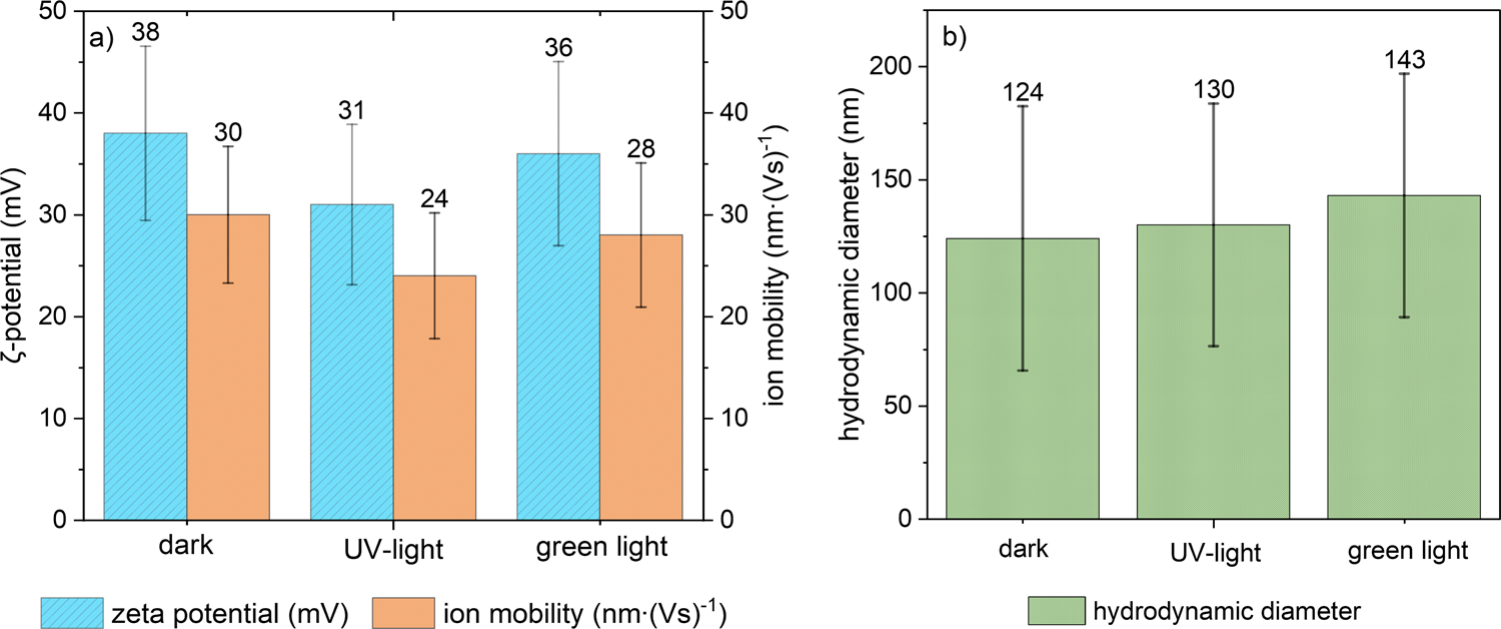
(a) ζ-Potential (blue, left axis) and ion mobility (orange, right axis) of aqueous solutions of 1 g·L^–1^ of **p­(iMe-AAPEAm-N­(CN)**_**2**_ **) P-2b** before irradiation (“dark”), after irradiation with UV light, and after irradiation with green light for 5 min; (b) hydrodynamic diameter of **P-2b** before irradiation (“dark”), after irradiation with UV light, and after irradiation with green light for 5 min. The numbers on top of the columns indicate the mean of the measured values. All measurements were conducted at ambient temperature.

Particle sizes of the aqueous polymer solution were measured by dynamic light scattering (DLS, Figure 7b). However, the rather high polydispersity of the sample hampers a cumulant analysis and leads to high deviations across the individual measurements. This observation suggests that the polymer is not dissolved molecularly or as small clusters of polymer chains but instead forms larger aggregates (diameter <200 nm), whose size does not change upon irradiation. Accordingly, the experiments do not show evidence for an effective modulation of the particle size via photoisomerization. Figure 7b presents the particle size for polymer **P-2b** before and after irradiation with UV- and green light, respectively, showing, within the precision of the measurements, no significant changes induced by the photoisomerization in either direction (Supplementary DLS data is provided in the Supporting Information, Figures S37–S39). The findings suggest that the permanent cationic charge of the pyrazolium group and its affinity to the low molar mass counterion, here dicyanamide, dominate the effective polarity/hydrophilicity of the dye moiety and its aqueous solution behavior, while the net effect of the photoisomerization is much smaller. Consequently, neither the solvation (and concomitantly, the hydrodynamic radius) nor the charge density and degree of counterion binding of the polymer change significantly upon irradiation.

Cryogenic scanning electron microscopy (cryo-SEM) is used to clarify the structure of **P-2b** in aqueous solution (*cf*. Supporting Information, Figures S40 and S41). Three specimens are imaged after plunge-freezing: the clear nonirradiated solution (I), the clear UV-irradiated solution (II), and the nonirradiated turbid dispersion (III). Specimen I is prepared by heating the heterogeneous polymer–water mixture above *T*_CLP_ before cooling the sample to ambient temperature. For specimen II, a fraction of the clear solution is irradiated with UV light. For specimen III, a fraction of the clear solution is rapidly cooled down with liquid nitrogen, inducing the phase separation. Yet, objects with dimensions correlating with the DLS findings are not observed in any case.

Given the amphiphilicity of **iMe-AAPEAm-N­(CN)**_**2**_ **2b** and its homopolymer **P-2b** due to the hydrophilic cationic site in the pyrazolium moiety and the overall quite hydrophobic azo dye chromophore, we wondered whether the structural similarity of the compounds to polymerizable surfactants (“surfmers”)^59^ and their polymers (“polysoaps”)^60^ is sufficient to induce surfactant-like behavior. Figure 8 illustrates the surface activity of both monomer and polymer in both isomers stated in aqueous solution. Monomer **2b** is indeed moderately surface active, with a maximum reduction of the surface tension to around 55 mN·m^–1^ at the solubility limit. Further, the monotonous decay of the surface tension with increasing concentration does not give a hint to the occurrence of a critical micelle concentration (*C*_MC_) or a critical aggregation concentration (*C*_AC_) up to the maximum solubility of around 3 g·L^–1^. Accordingly, the amphiphilicity of monomer **2b** is not sufficient to make it a true surfmer, but it may be rather considered to be a polymerizable hydrotrope.^61,62^

**Figure 8:**
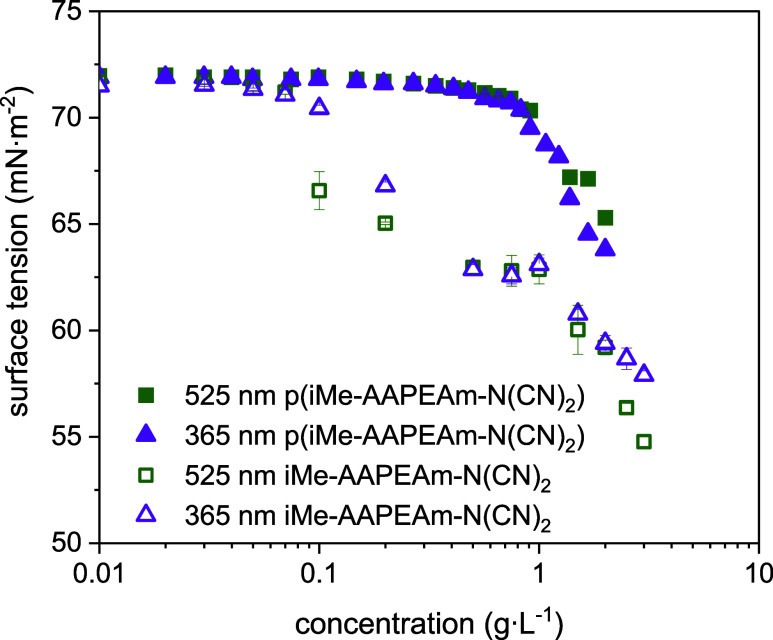
Surface tension of the aqueous solutions of monomer **iMe-AAPEAm-N­(CN)**_**2**_ (**2b**) (hollow symbols) and its polymer **P-2b** (full symbols) vs concentration at 20 ± 0.1 °C. The sample after 10 min green light irradiation (525 nm, majority *E*-isomer) is shown as green squares. The sample after 10 min UV-light irradiation (365 nm, majority *Z*-isomer) is shown as purple triangles.

The even lower, very weak surface activity of the corresponding homopolymer, **P-2b**, parallels the difference in behaviors between surfmers and the respective polysoaps, in particular for the structurally most closely related cationic polysoaps of the tail-end type.^63,64^ The findings may be explained by the specific chemical structure of the monomer, in which hydrophobic alkyl chains are virtually missing despite the inherent amphiphilic character of the chromophore.^60^ Also, photoisomerization upon irradiation hardly changes the surface activity. The observed differences in surface tension reduction between the *E*- and *Z*-isomers are marginal, if significant at all, for both the monomer and the polymer.

## Conclusions

A set of cationic arylazopyrazolium-based monomers and polymers with differing anions is synthesized starting from the previously described nonionic precursor monomer AAPEAm.^21^ The low melting point of 44 °C of one of the derivatives, namely, of the dicyanamide salt, qualifies it as a polymerizable ionic liquid, and its homopolymer may be considered as a polyelectrolyte of the class of polymerized ionic liquids. The various polycations are water-soluble, with an upper critical solution temperature (UCST) with clearing points of around 60–70 °C. While UCST behavior has been known for certain hydrophobic polyelectrolytes,^65^ a more intriguing aspect is that once dissolved at elevated temperatures, the polymer does not phase-separate again upon conventional cooling, even at temperatures as low as 4 °C. Only rapid cooling with liquid nitrogen to temperatures near the freezing point induces polymer precipitation, resulting in a suspension that sediments over several hours. Although the polymer can be dissolved and reprecipitated repeatedly, rendering it thermoresponsive by definition, the kinetic stabilization of the visually dissolved polymerized ionic liquid significantly limits its applicability for conventional uses, such as sensors or shape-memory materials. Furthermore, comparative ^1^H NMR studies in DMSO–D_6_, MeOD-D_4_, and D_2_O at ambient temperatures show that the polymer apparently forms aggregates in water with low molecular mobility, suppressing its ^1^H signals. In combination with studies of the ζ-potential, dynamic light scattering, surface activity, and cryogenic-SEM, this suggests that some kind of self-assembly of the polymer takes place in water, notwithstanding the fact that the amphiphilicity of the monomer is not sufficient to qualify them as true surfactants but rather to be hydrotropes.

The introduction of a positive charge on the five-membered heterocycle virtually does not affect the dye’s spectral properties compared to the nonionic precursor AAPEAm. The chromophore retains well-separated absorption maxima for the *E*-and *Z*-isomers, allowing nearly quantitative and reversible switching between the two states by irradiation with UV- and green light, respectively. Polymerization and the change of the solvent from water to methanol have only minor effects on the absorption spectra. This suggests that while the microphase separation indicated by NMR may affect the polymer as a whole, the photoisomerization of the azopyrazolium group remains mostly unaffected. However, the thermal half-lives of the *Z*-state achieved in the photostationary state of both the monomer and the polymer, specifically in water, are reduced to a few hours only at room temperature. This is notably shorter than their half-lives in methanol, which compare well with those of uncharged arylazopyrazole monomers and polymer analogues. Nevertheless, the half-life in aqueous solution may still be sufficient for most experiments to be conducted with a single irradiation at the start without the need to apply continuous irradiation.

## Supplementary Material



## Data Availability

The data underlying this study are available in the published article and its Supporting Information.

## Notes

Detailed synthetic procedures and analytical data; ^1^H and ^13^C NMR spectra, FT-IR spectra, and EDS spectra of monomers and polymers; comparison of the UV–vis spectra of the nonirradiated and UV-irradiated precursor monomer and quaternized monomers in methanol and in water; visual appearance of aqueous polymer solutions at ambient temperatures; ^1^H NMR spectra of the quaternized monomer in methanol before irradiation (dark annealed), after irradiations at 365 nm and at 525 nm, respectively, in the photostationary states; determination of the half-lives of the quaternized monomer and polymer; particle size distribution by DLS; cryo-SEM micrographs of plunge-frozen aqueous polymer solutions and EDS atom count maps (PDF)
