# Supplementary material for: Photoresponsive Polycations Bearing an Arylazopyrazolium Dye
Source: ACS Omega. 2026 Mar 17;11(12):19758–68. doi: 10.1021/acsomega.6c00099 (PMC13044631; doi:10.1021/acsomega.6c00099)
Supplement: Supplementary file 1 [file ao6c00099_si_001.pdf]

Supporting Information of:

Photo-responsive Polycations Bearing an Arylazopyrazolium Dye

René Steinbrecher, [a] Martin Reifarth, [a, b] Jiayin Yuan, [c] Christine M. Papadakis, [d] Peter Müller-Buschbaum, [d] Andreas Taubert, [a] and André Laschewsky [a, b]*

a Institute of Chemistry, University of Potsdam, 14476 Potsdam-Golm, Germany.
E-mail: laschews@uni-potsdam.de.

b Fraunhofer Institute for Applied Polymer Research IAP, 14476 Potsdam-Golm, Germany

c Department of Chemistry, Stockholm University, 10691 Stockholm, Sweden

d TUM School of Natural Sciences, Department of Physics, Technical University of Munich, 85748 Garching, Germany

Elemental analysis by energy-dispersive X-ray spectroscopy (EDS)

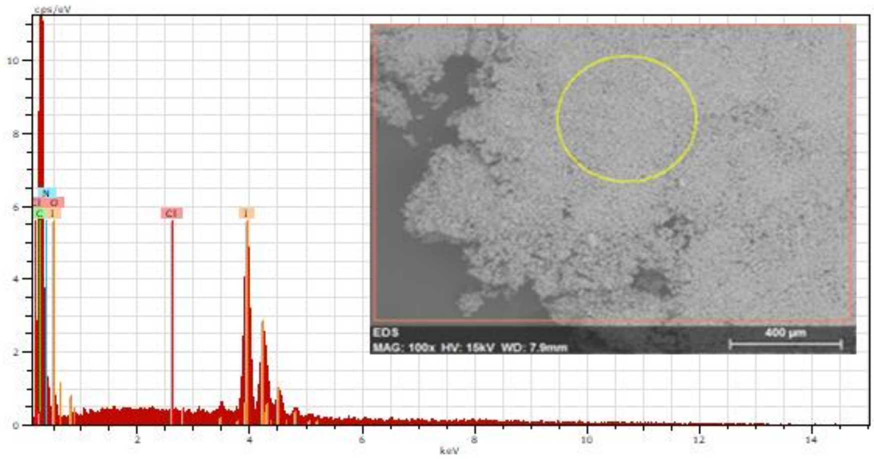

Figure S1: EDS spectrum of monomer iMe-AAPEAm-I, confirming the presence of iodide after the quaternization reaction with CH_3I.

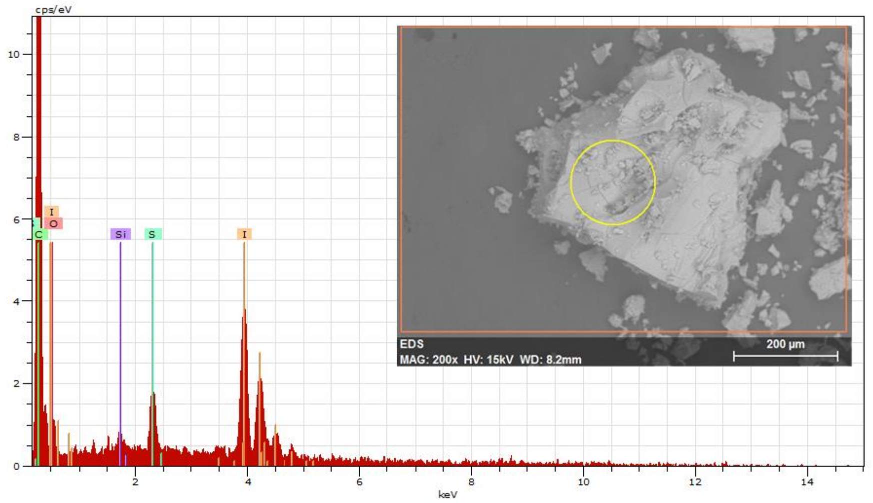

Figure S2: EDS spectrum of p(iMe-AAPEAm-I) confirming the presence of iodide in the polymer.

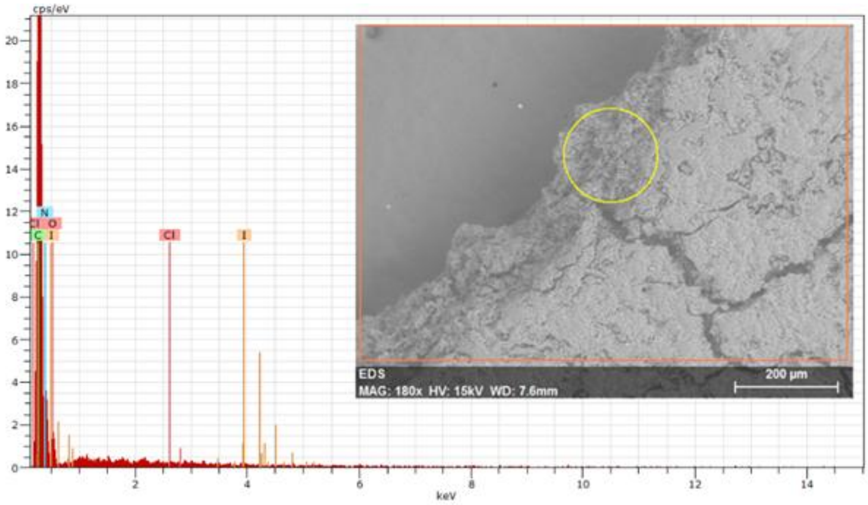

Figure S3: EDS spectrum of monomer iMe-AAPEAm-N(CN)$_2$ confirming the absence of iodide after the ion exchange with AgN(CN)$_2$.

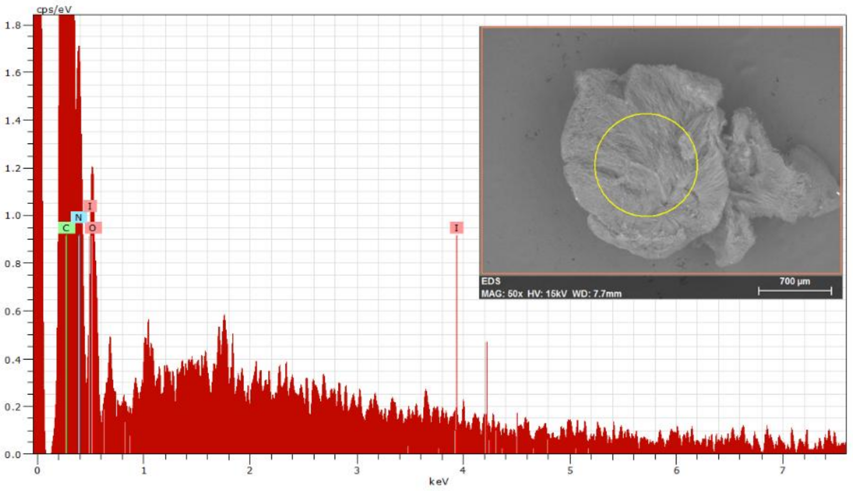

Figure S4: EDS spectrum of polymer p(iMe-AAPEAm-N(CN)$_2$) confirming the absence of iodide in the polymer.

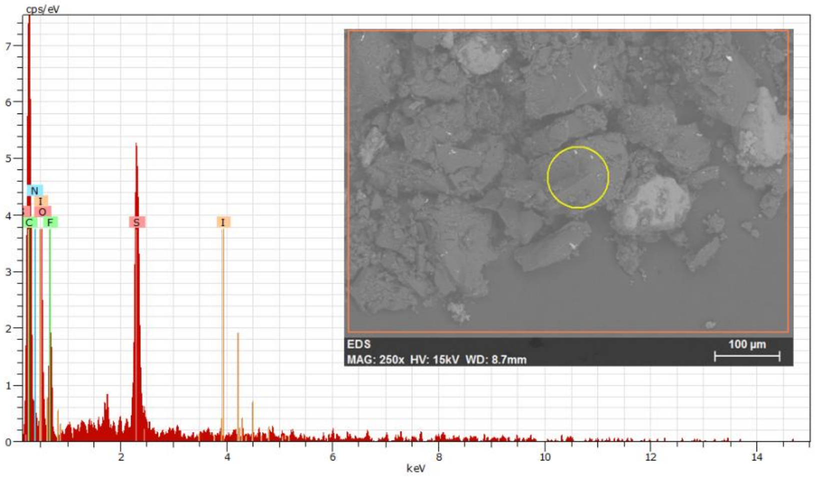

Figure S5: EDS spectrum of p(iMe-AAPEAm-CF$_3$SO$_3$) confirming the absence of iodide in the polymer.

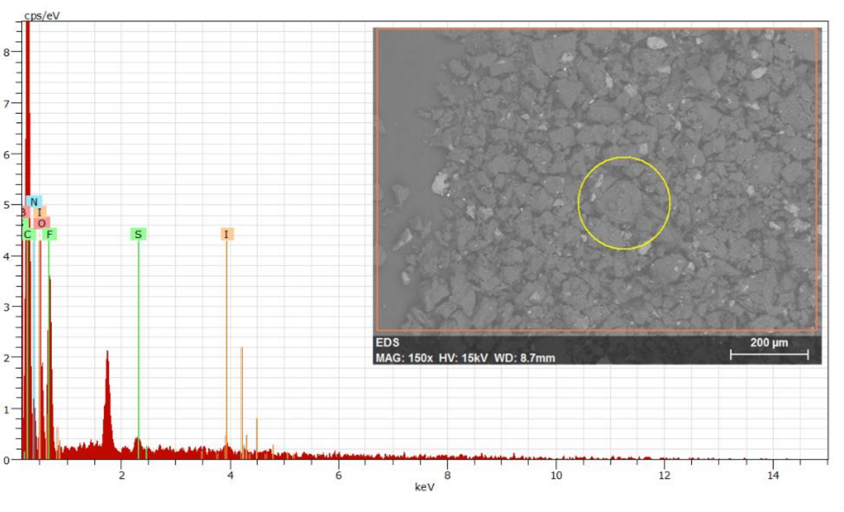

Figure S6: EDS spectrum of p(iMe-AAPEAm-BF_4) confirming the absence of iodide the polymer.

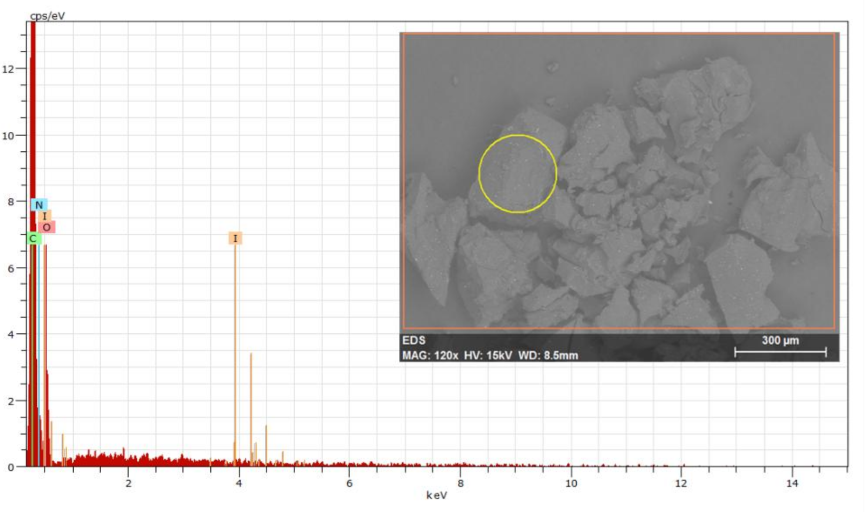

Figure S7: EDS spectrum of p(iMe-AAPEAm-NO_3) confirming the absence of iodide in the polymer.

Syntheses

Quaternization of AAPEAm:

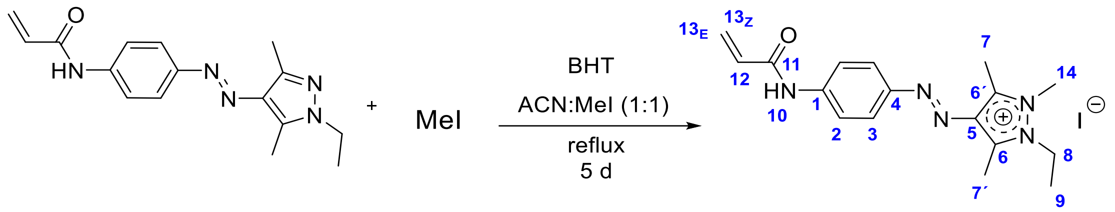

Scheme S1: Quaternization of AAPEAm with CH_3I (MeI)

Adapting a literature protocol,[1] the precursor monomer AAPEAm (4.0 g, 13.45 mmol, 1 Eq.) was dissolved in the minimum volume necessary of dry MeCN and CH_3I (1:1v) (100 mL in total) in a dry flask. BHT (stabilizer, 15-20 mg) was added to the solution. The reaction mixture was stirred at reflux for 5 d (monitoring the reaction progress with TLC, ethyl acetate : *n*-hexane (EA : *n*H) (3:1v)). The solution turns slightly turbid after 2 to 3 d,. After total conversion of AAPEAm (determined by TLC), the reaction mixture was cooled to room temperature and reduced to around 20 mL in reduced pressure. Diethyl ether was added to the cooled mixture to precipitate the product further, and the suspension was kept in the refrigerator overnight. Then, the precipitate was filtered off and thoroughly washed excessively with distilled diethyl ether. After drying over night at 45°C, the product was received as an orange solid (yield 5.6 g, 95%).

^{1}H NMR (400 MHz, DMSO-D_6, 298K): δ = 10.54 (s, 1H, 10-H), 8.15-7.71 (s, 4 H, 2+3-H), 6.46 (dd, 1 H 12-H), 6.35 (d, 1 H ,13_Z-H), 5.9 (d, 1 H, 13_E-H), 4.60 (q, 2 H, 8-H), 4.08 (s, 3 H, 14-H), 2.76 (s, 6 H, 7-H), 1.38 (t, 3 H, 9-H) ppm.

^{13}C NMR (100 MHz, DMSO-D_6, 298K): δ =164.00 (11-C), 148.31 (4-C), 142.85 (1-C), 142.21, 140.92 (6-C), 132.97 (5-C), 131.97 (13-C), 128.36 (12-C), 123.94, 120.04 (2+3-C), 42.58 (8-C), 34.43 (14-C), 14.19, 11.16 (7-C), 10.80 (9-C) ppm.

TLC (*n*-hexane:EA 1:3): R_f = 0.00 (stays at the starting point)

T_{melt} (by DSC): 199 °C

The presence of iodide was verified by EDS

ATR-FTIR spectrum (powder):

**wavenumber (cm⁻¹)**	**putative assignment**	**group**
3457	*v*(N-H)	amine
3025	*v*(C-H)	alkene
1679	*v*(C=O)	amide
1597	*v*(-C-C) in ring	aromatic ring

Synthesis of $AgN(CN)_2$

$$NaN(CN)_2 + AgNO_3 \xrightarrow[\text{rt, 0.5 h}]{H_2O} AgN(CN)_2$$

Scheme S2: Ion exchange between $NaN(CN)_2$ and $AgNO_3$ in water to precipitate the product $AgN(CN)_2$.

Adapting a literature procedure,[2] a solution of 9.54 g (1 Eq., 56.16 mmol) of $AgNO_3$ in 60 mL of distilled water was added dropwise to the solution of 5.00 g (1 Eq., 56.16 mmol) of sodium dicyanamide in 120 mL of distilled water under stirring. After 30 min, the precipitate formed was filtered off and rinsed with 30 mL of distilled water. The precipitate was dried at 70 °C until no more weight loss was observed, to obtain silver dicyanamide $AgN(CN)_2$ as a white solid (9.55 g, 98 %).

Ion exchange reaction of iMe-AAPEAm-I with $AgN(CN)_2$

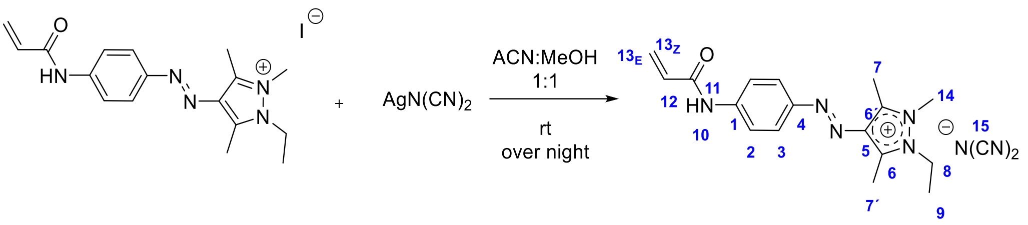

Scheme S3: Ion exchange of iMe-AAPEAm-I (**2a**) with $AgN(CN)_2$ to produce the ionic liquid (IL) monomer iMe-AAPEAm-$N(CN)_2$ (**2b**).

Adapting a literature procedure,[1] in a round bottom flask 2.00 g (1 Eq., 4.55 mmol) of iMe-AAPEAm-I was dissolved in a mixture of ACN and MeOH (1:1v). Silver dicyanamide (2.38 g, 13.66 mmol, 3 Eq.) was added in excess into the above solution and stirred overnight. After 12 h this solution was filtered through a fritted glass that is covered with AlOx basic and the solution was evaporated to dryness yielding the desired product iMe-AAPEAm-$N(CN)_2$ as an orange solid. (1.67 g, 97 %)

^{1}H NMR (400 MHz, DMSO-D$_6$, 298K): δ = 10.54 (s, 1H, 10-H), 8.15-7.71 (s, 4 H, 2+3-H), 6.48 (dd, 1 H 12-H), 6.34 (d, 1 H ,13$_Z$-H), 5.86 (d, 1 H, 13$_E$-H), 4.61 (q, 2 H, 8-H), 4.08 (s, 3 H, 14-H), 2.75 (s, 6 H, 7-H), 1.38 (t, 3 H, 9-H) ppm.

^{13}C NMR (100 MHz, DMSO-D$_6$, 298K): δ =164.00 (11-C), 148.31 (4-C), 142.85 (1-C), 142.21, 140.92 (6-C), 132.97 (5-C), 131.97 (13-C), 128.36 (12-C), 123.94, 120.04 (2+3-C), 119.53 (C-15), 42.49 (8-C), 34.24 (14-C), 14.12, 11.06 (7-C), 10.72 (9-C) ppm.

TLC (*n*H : EA 1:3): R_f = 0.00 (stays at the starting point)

T_{melt} (by DSC): 44 °C

Mass: Calculated (without counter ion): 312.40 g·mol^{-1}, found 312.25 (M^+)

Elemental analysis:

Calculated	Found
C: 60.30 %	C: 59.05 %
H: 5.86 %	H: 5.85 %
N: 29.61 %	N: 29.48 %

The absence of iodide was verified by EDS

ATR-FTIR spectrum (powder):

**wavenumber (cm^{-1})**	**putative assignment**	**group**
3505	*v*(N-H)	amine
3012	*v*(C-H)	alkene
2242	v(-C≡N)	cyanide
1679	*v*(C=O)	amide
1597	*v*(-C-C) in ring	aromatic ring

Polymerization

iMe-AAPEAm-N(CN)2 and the other pyrazolium monomers with different counter ions are polymerized by free radical polymerization (Scheme S4). Following the scheme, the polymerization procedure for p(iMe-AAPEAm-N(CN)2) **P-2b** is described.

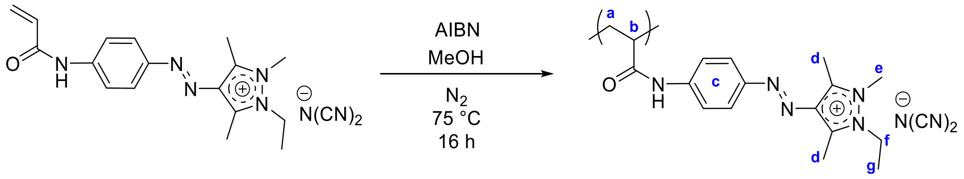

iMe-AAPEAm-N(CN)2 **p(iMe-AAPEAm-N(CN)2)**

Scheme S4: Free radical polymerization of iMe-AAPEAm-N(CN)2 (**2b**) in DMSO or MeOH with AIBN as thermal initiator.

In a typical procedure, iMe-APPEAm-N(CN)2 (**2b**, 1.0 g, 2.6 mmol, 100 mol-%) and AIBN (21.7 mg, 0.1 mmol, 5 mol %) were placed in a dry Schlenk tube, and dissolved in MeOH to prepare a 2 mol·L^{-1} solution (regarding the monomer and initiator). The tube was sealed with a septum, and the mixture was purged with a gentle nitrogen stream for 15 min to remove the oxygen. The solution was then immersed into a preheated 75 °C hot oil bath and stirred for 7 h. Conversion was monitored by ^{1}H NMR, observing the decay of the acrylic proton signal at around 5.8 ppm. Since the polymerization of iMe-AAPEAm-N(CN)2 seems to be hindered, a higher amount of AIBN was needed. The polymerization was stopped by removing the septum and exposing the mixture to air, and cooling the vessel rapidly to r.t. The cooled mixture was precipitated into a 10-fold volume of a mixture of diethyl ether and THF (1:1v), and the precipitated polymer was collected via filtration. The process was repeated until no monomer signal was detected in the ^{1}H NMR spectrum. The polymer was dried in the drying oven at

70°C. At last, the polymer is dissolved in water and freeze dried to yield an orange solid. (760 mg)

^{1}H NMR (400 MHz, MeOD-D$_4$, 298K): δ = 7.64 (s, 4 H, c-H), 4.46 (s, 2 H, f-H), 3.96 (s, 3 H, e-H), 2.62 (s, 6 H, d-H), 1.92, 1.36, 1.19 (6 H, b, g, a-H) ppm.

Aqueous SEC (poly(ethylene glycol) standards):

M_n^{app} = 9.20·10^3 g·mol^{-1}, M_w^{app} = 1.27·10^3 g·mol^{-1}, Đ = 1.4, DP = 25 (based on the M_n and the molar mass M_r of the monomer with counter-ion).

The absence of iodide was verified by EDS

ATR-FT-IR spectrum (powder):

**wavenumber (cm^{-1})**	**putative assignment**	**group**
3401	*v*(N-H)	amine
3027	*v*(C-H)	alkene
2235	*v*(-C≡N)	cyanide
1679	*v*(C=O)	amide
1593	*v*(-C-C) in ring	aromatic ring

Further ion exchange reactions and free radical polymerizations

The anion exchange by various silver salts (NO_3^-, $CF_3SO_3^-$, BF_4^-) were conducted analogously to the ion exchange described with Ag(NCN)$_2$. The homopolymerizations of the monomers **2a**, **2c**, **2d**, and **2e** were conducted following the polymerization procedure described for iMe-AAPEAm-N(CN)$_2$ (**2b**).

NMR spectra

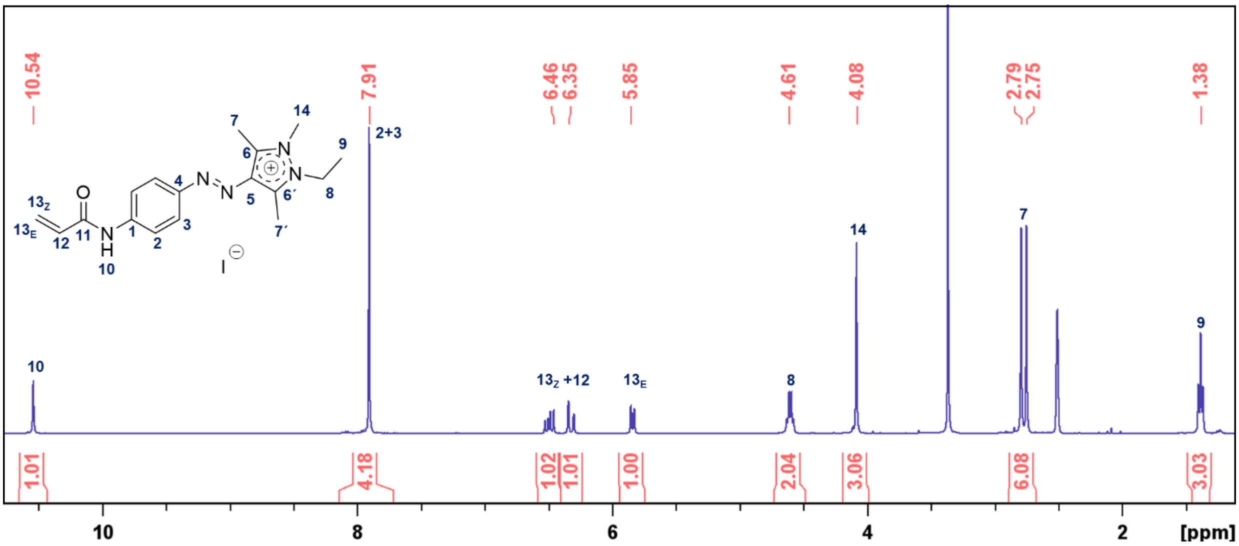

Figure S8: ^{1}H NMR of monomer iMe-AAPEAm-I (**2a**) (400 MHz, DMSO-D6, 298 K): Blue numbers connect protons with the corresponding signal. Chemical shifts are given in ppm.

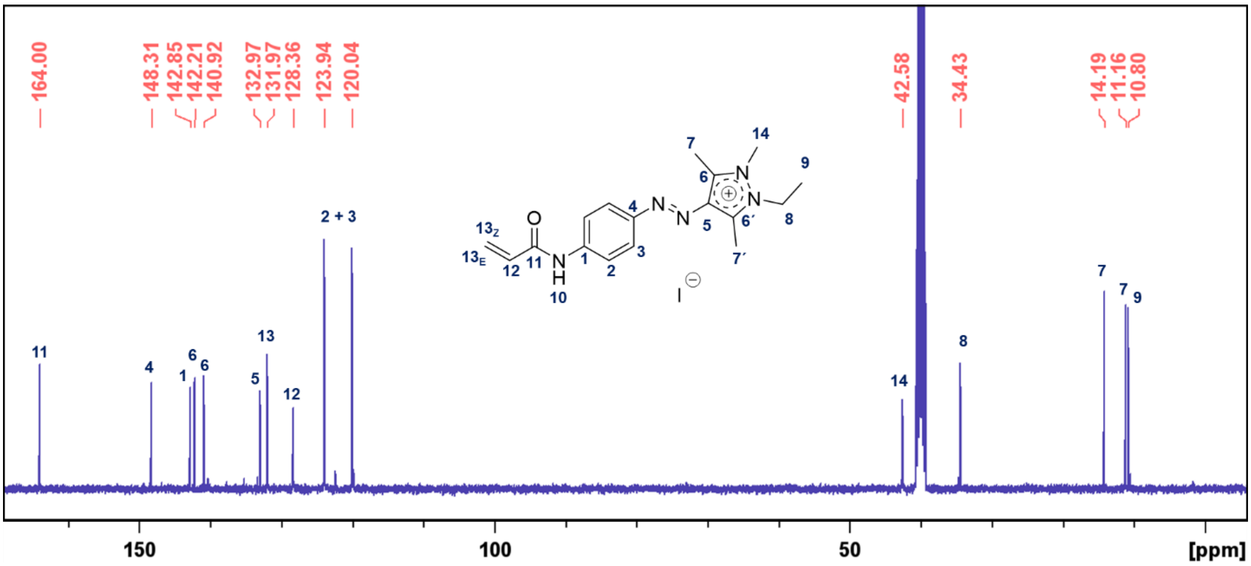

Figure S9: ^{13}C NMR of monomer iMe-AAPEAm-I (**2a**) (100 MHz, DMSO-D6, 298 K): Blue numbers connect carbon atoms with the corresponding signal. Chemical shifts are given in ppm.

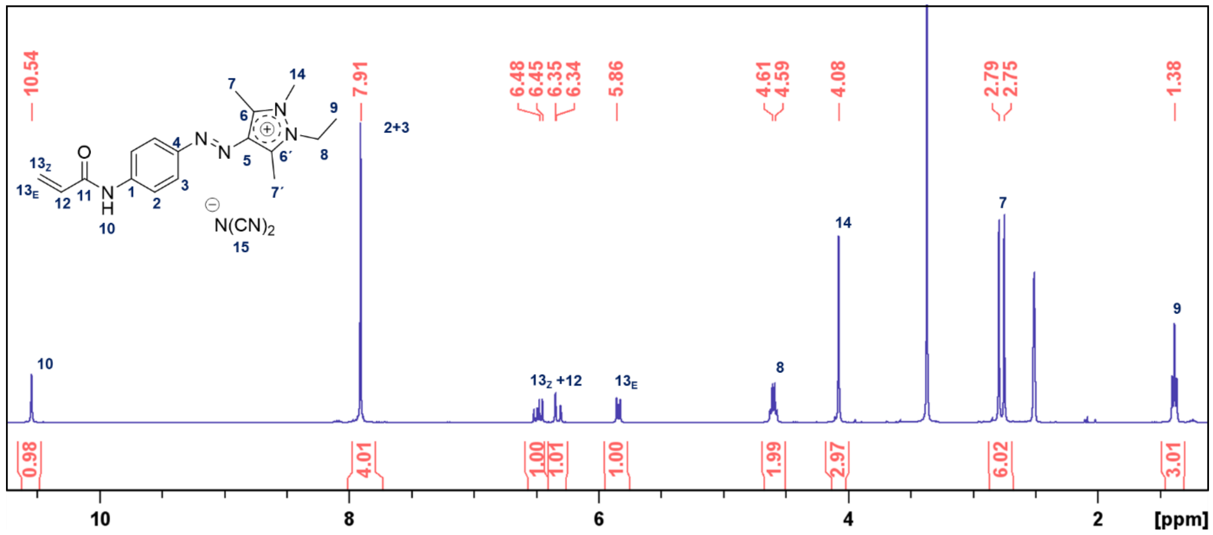

Figure S10: ^{1}H NMR of monomer iMe-AAPEAm-N(CN)$_2$ (**2b**) (400 MHz, DMSO-D$_6$, 298 K): Blue numbers connect protons with the corresponding signal. Chemical shifts are given in ppm.

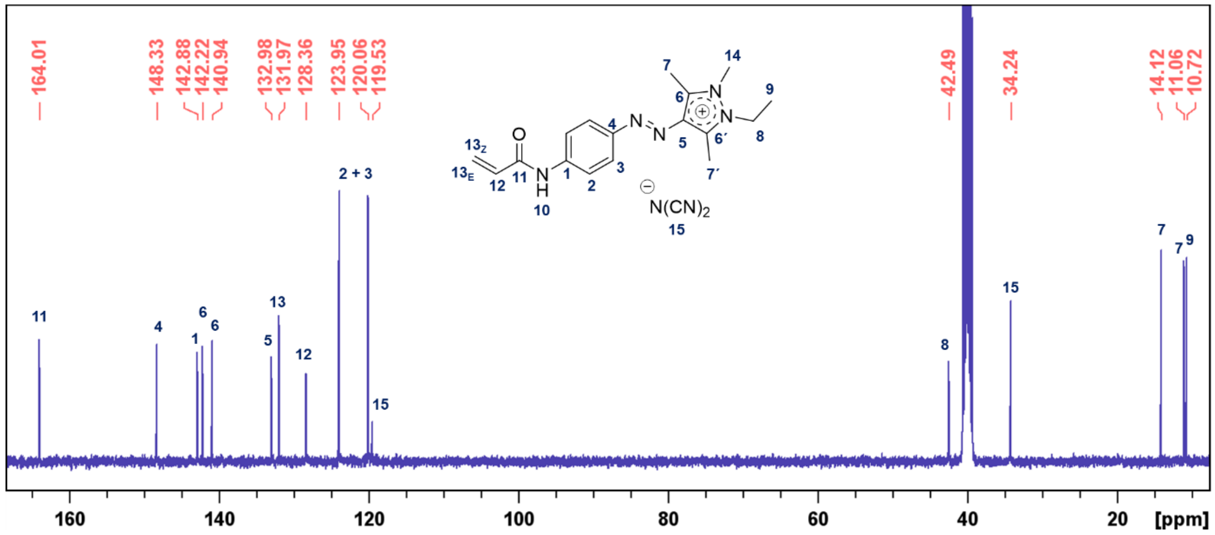

Figure S11: ^{13}C NMR of monomer iMe-AAPEAm-N(CN)$_2$ (**2b**) (100 MHz, DMSO-D$_6$, 298 K): Blue numbers connect carbon atoms with the corresponding signal. Chemical shifts are given in ppm.

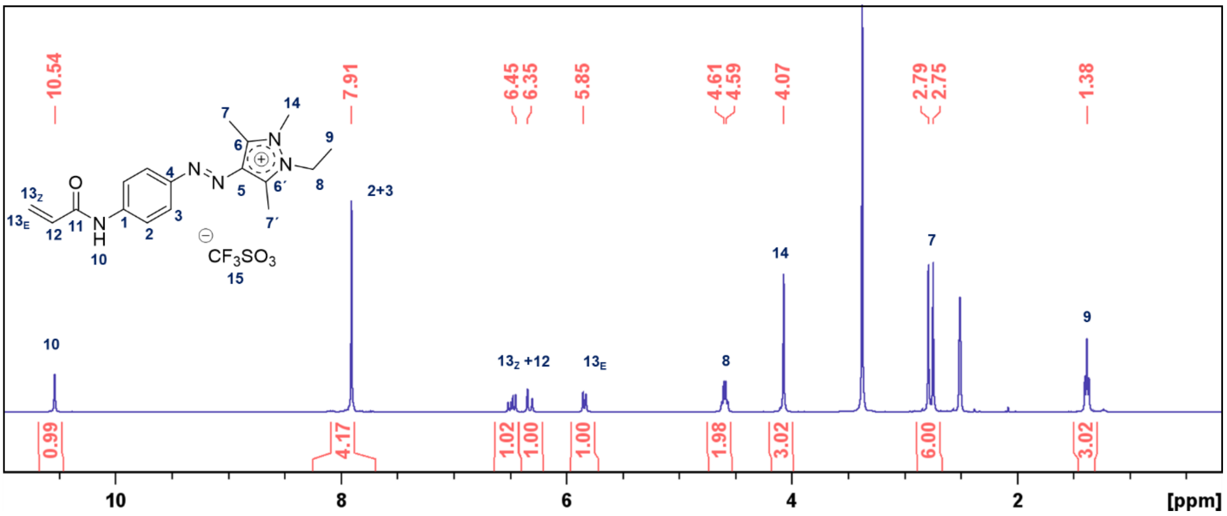

Figure S12: ^{1}H NMR of monomer iMe-AAPEAm-CF_3SO_3 (**2d**) (400 MHz, DMSO-D_6, 298 K): Blue numbers connect protons with corresponding signal. Chemical shifts are given in ppm.

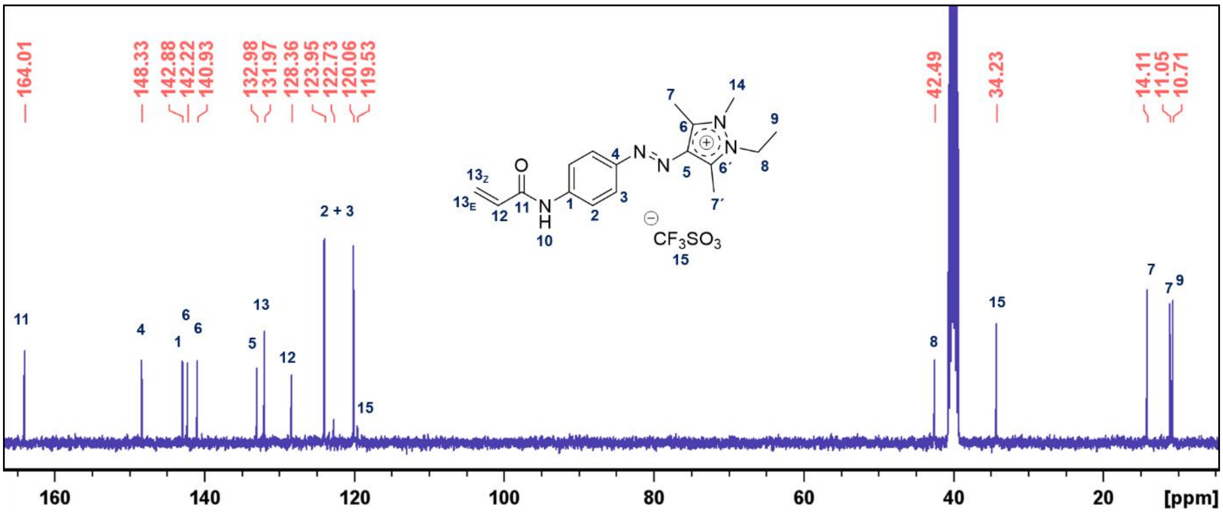

Figure S13: ^{13}C NMR of monomer iMe-AAPEAm-CF_3SO_3 (**2d**) (100 MHz, DMSO-D_6, 298 K): Blue numbers connect carbon atoms with the corresponding signal. Chemical shifts are given in ppm.

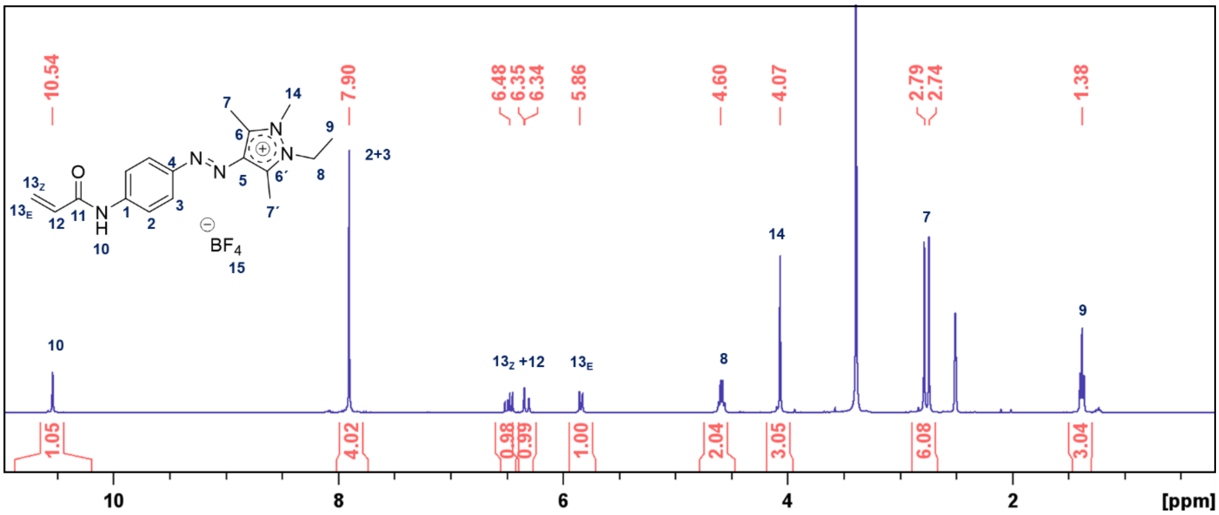

Figure S14: ^{1}H NMR of monomer iMe-AAPEAm-BF$_4$ (**2e**) (400 MHz, DMSO-D$_6$, 298 K): Blue numbers connect protons with the corresponding signal. Chemical shifts are given in ppm.

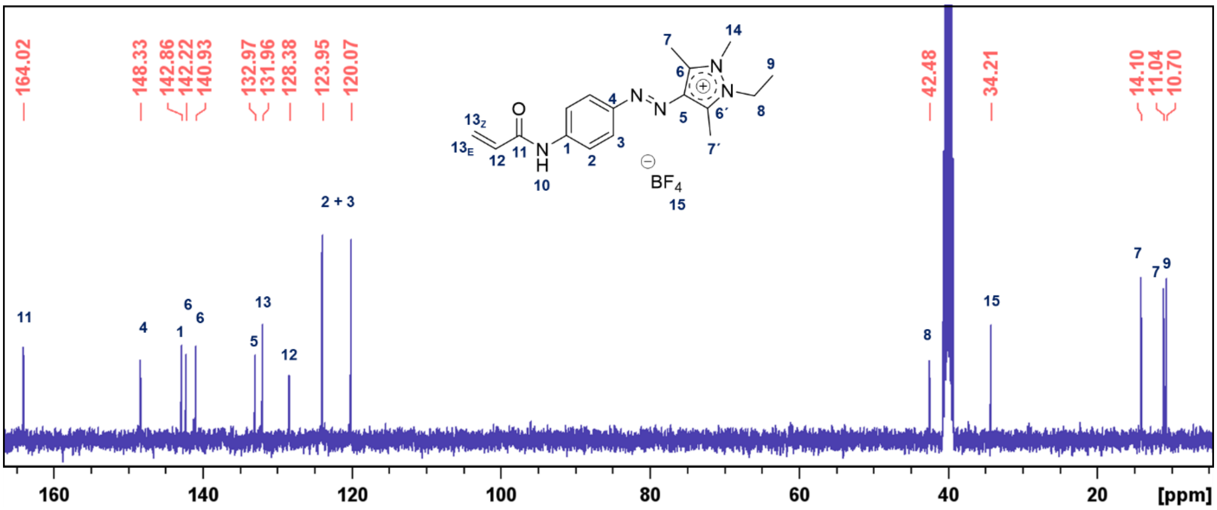

Figure S15: ^{13}C NMR of monomer iMe-AAPEAm-BF$_4$ (**2e**) (100 MHz, DMSO-D$_6$, 298 K): Blue numbers connect carbon atoms with the corresponding signal. Chemical shifts are given in ppm.

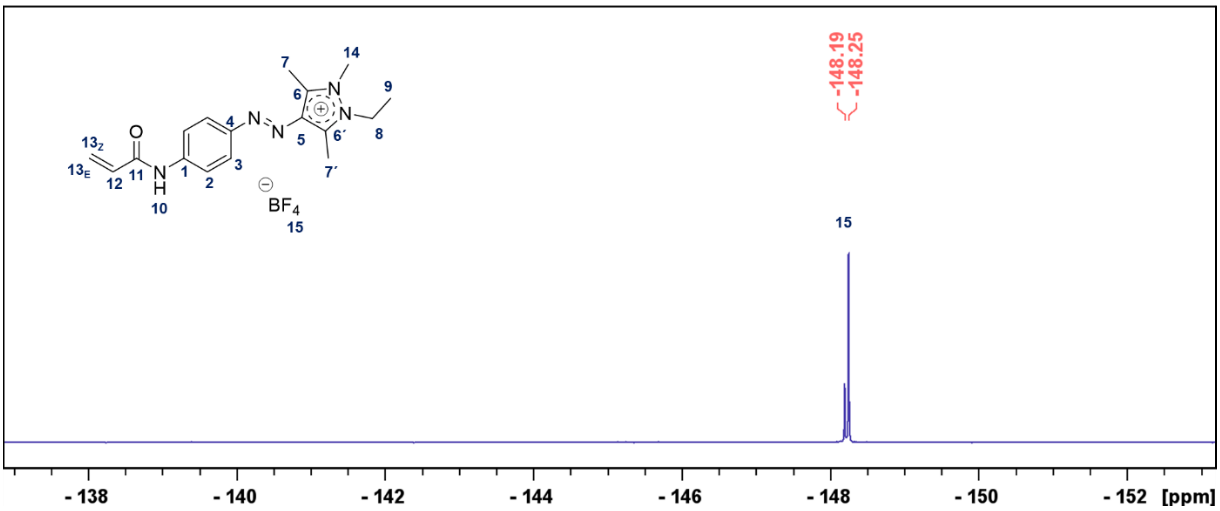

Figure S16: ^{19}F NMR of monomer iMe-AAPEAm-BF_4 (**2d**) (376 MHz, DMSO-D_6, 298 K): Blue numbers connect fluorine atoms with the corresponding signal. Chemical shifts are given in ppm. The split signal is due to the boron isotope effect.

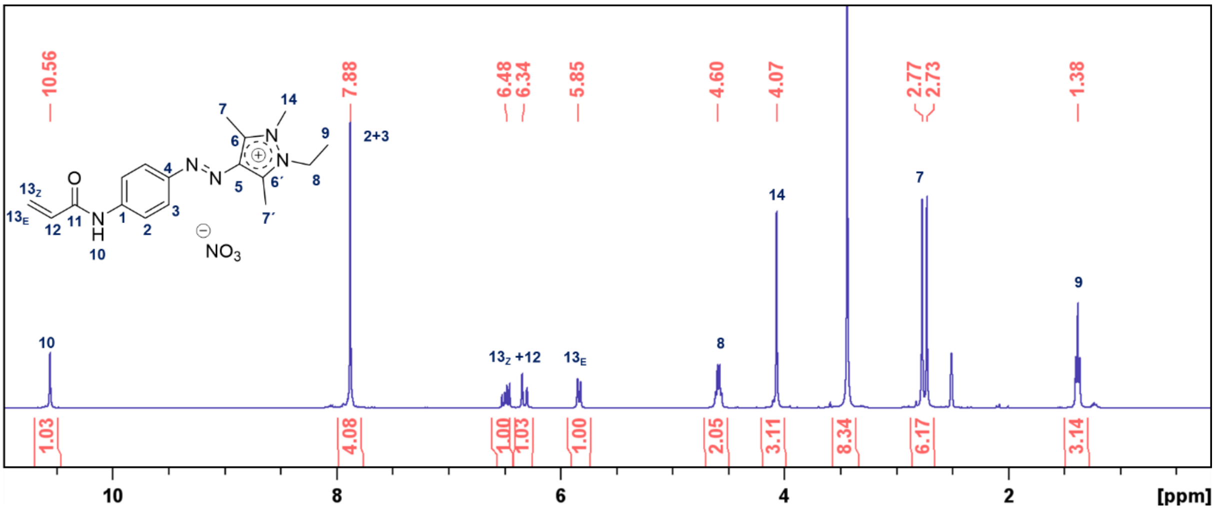

Figure S17: ^{1}H NMR of monomer iMe-AAPEAm-NO_3 (**2c**) (400 MHz, DMSO-D_6, 298 K): Blue numbers connect protons with the corresponding signal. Chemical shifts are given in ppm.

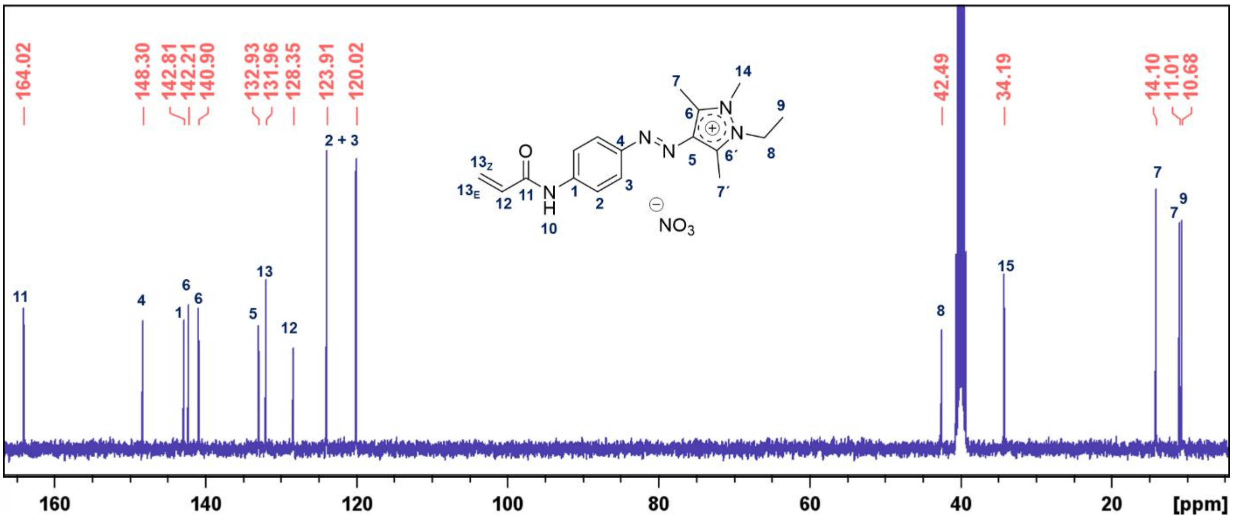

Figure S18: ^{13}C NMR of monomer iMe-AAPEAm-NO_3 (**2c**) (100 MHz, DMSO-D_6, 298 K): Blue numbers connect carbon atoms with the corresponding signal. Chemical shifts are given in ppm.

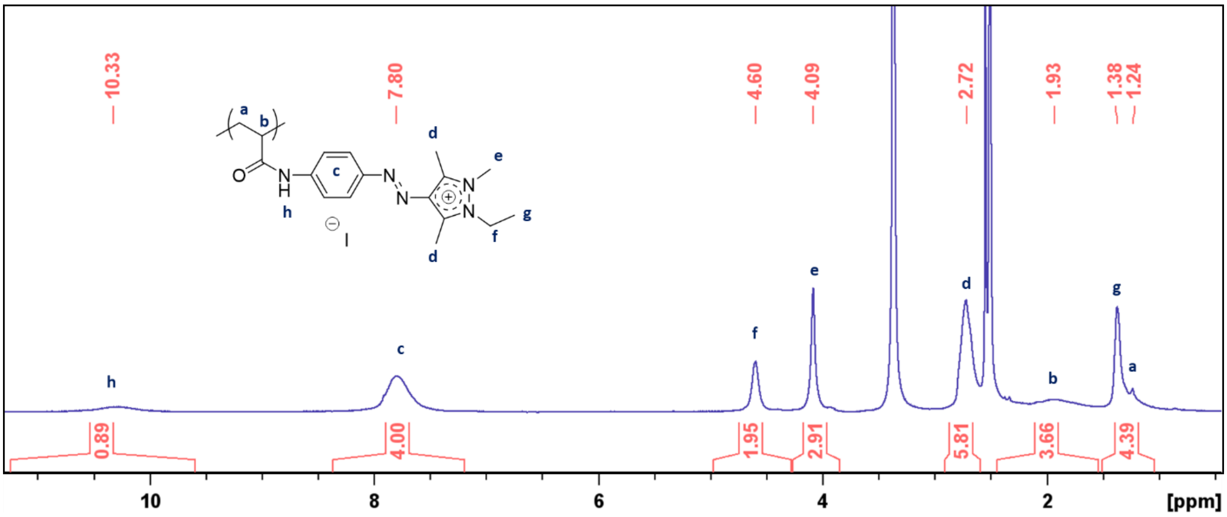

Figure S19: ^{1}H NMR of polymer p(iMe-AAPEAm-I) (**P-2a**) (400 MHz, DMSO-D_6, 298K): Blue numbers connect protons with the corresponding signal. Chemical shifts are given in ppm.

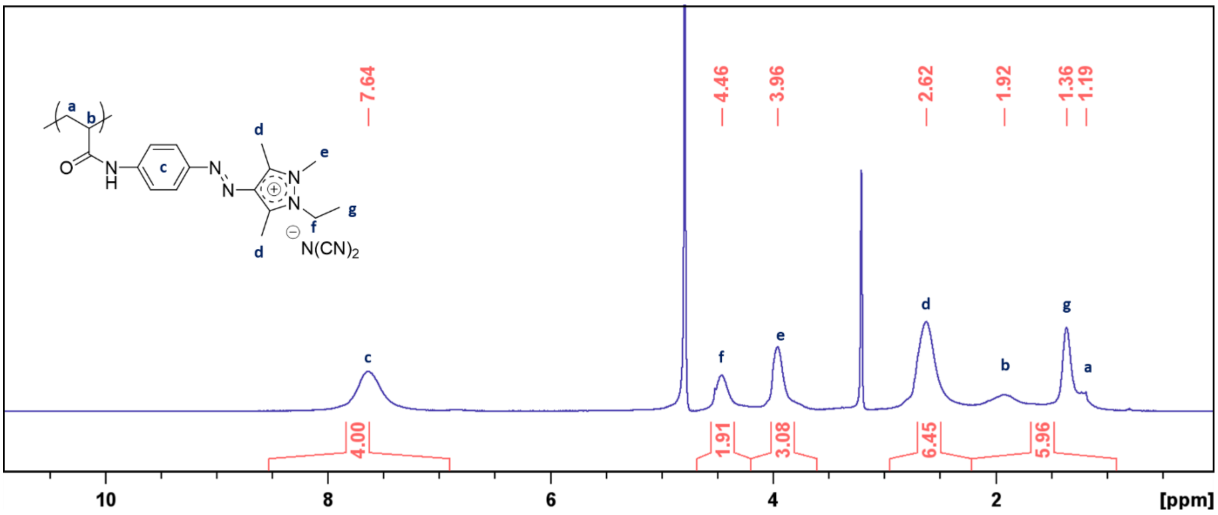

Figure S20: ^{1}H NMR of polymer p(iMe-AAPEAm-N(CN)$_2$) (**P-2b**) (400 MHz, MeOD-D$_4$, 298 K): Blue numbers connect protons with the corresponding signal. Chemical shifts are given in ppm.

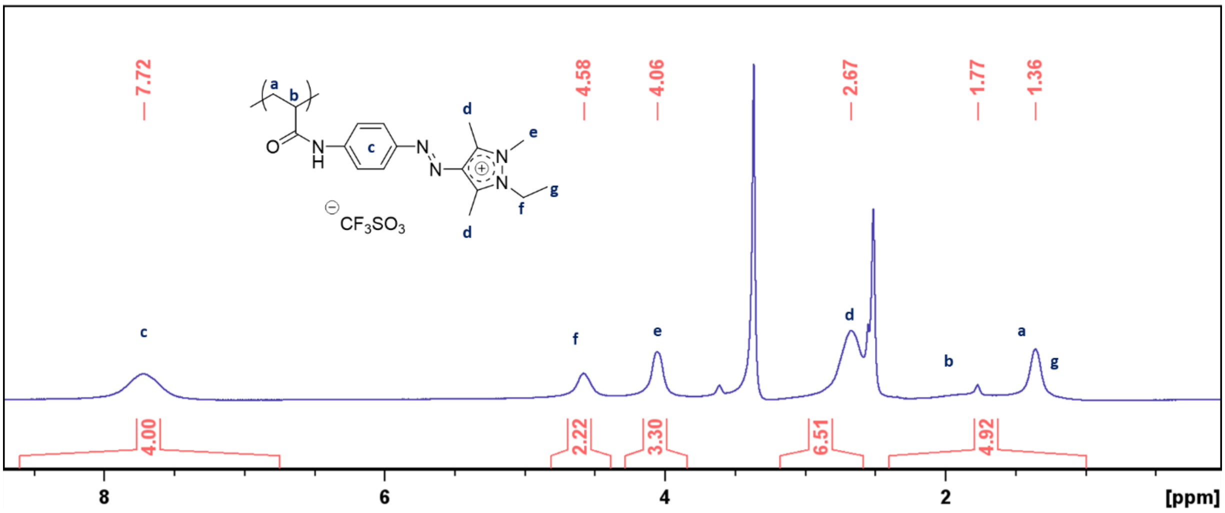

Figure S21: ^{1}H NMR of polymer p(iMe-AAPEAm-CF$_3$SO$_3$) (**P-2d**) (400 MHz, DMSO-D$_6$, 298 K): Blue numbers connect protons with the corresponding signal. Chemical shifts are given in ppm.

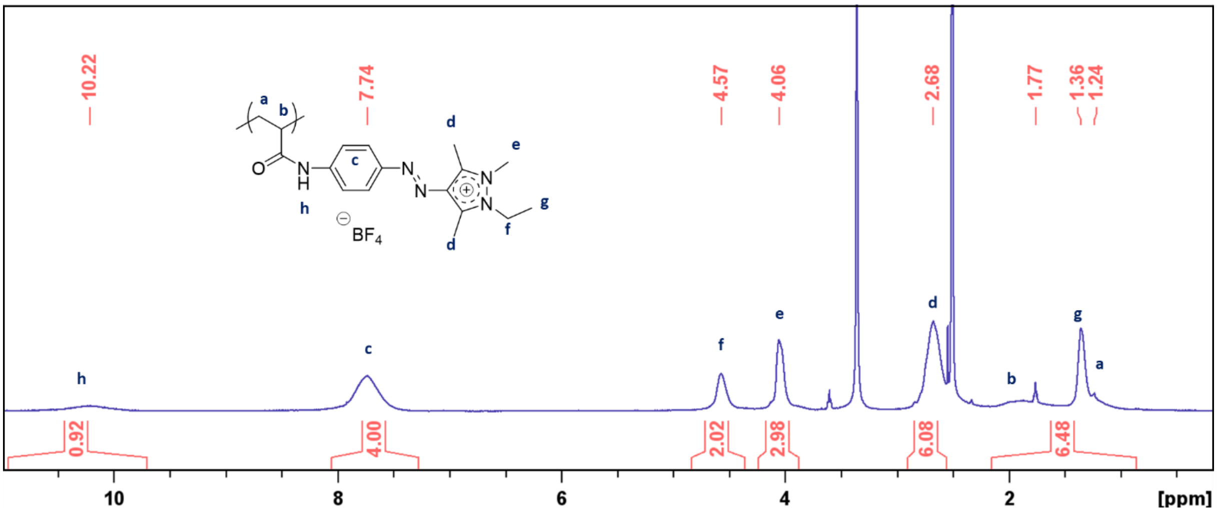

Figure S22: ^{1}H NMR of polymer p(iMe-AAPEAm-BF_4) (**P-2e**) (400 MHz, DMSO-D_6, 298 K): Blue numbers connect protons with the corresponding signal. Chemical shifts are given in ppm.

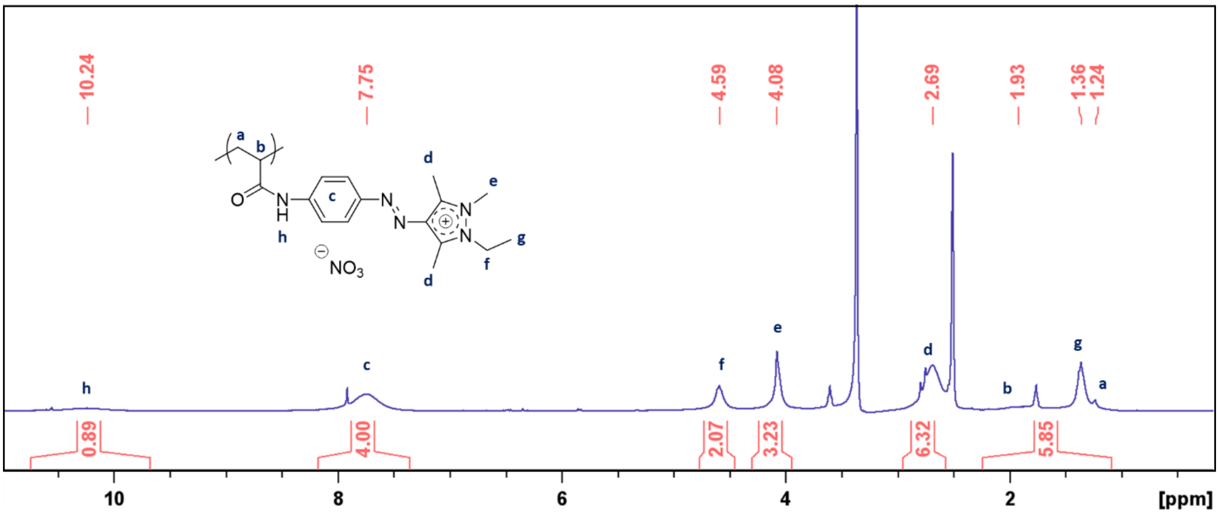

Figure S23: ^{1}H NMR of polymer p(iMe-AAPEAm-NO_3) (**P-2c**) (400 MHz, DMSO-D_6, 298 K): Blue numbers connect protons with the corresponding signal. Chemical shifts are given in ppm.

IR spectra

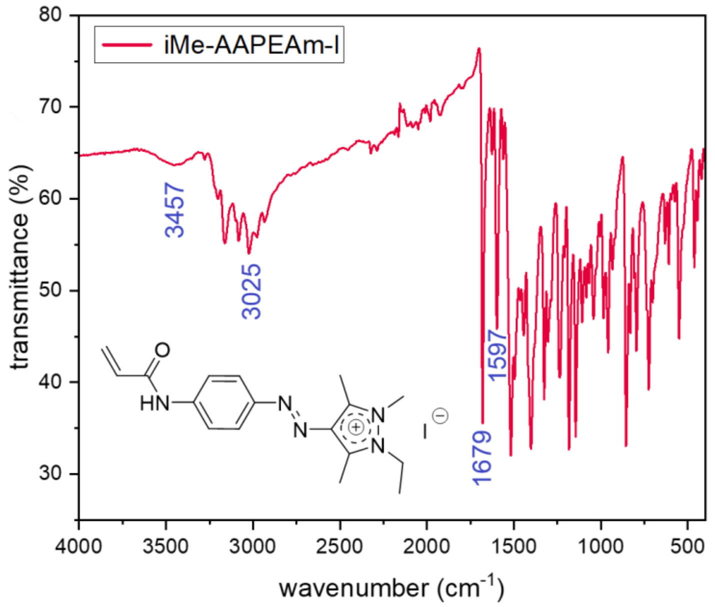

Figure S24: FT-IR spectra of monomer iMe-AAPEAm-I (**2a**) (ATR, 298K): Blue numbers indicate wavenumbers with corresponding functional groups or bonds.

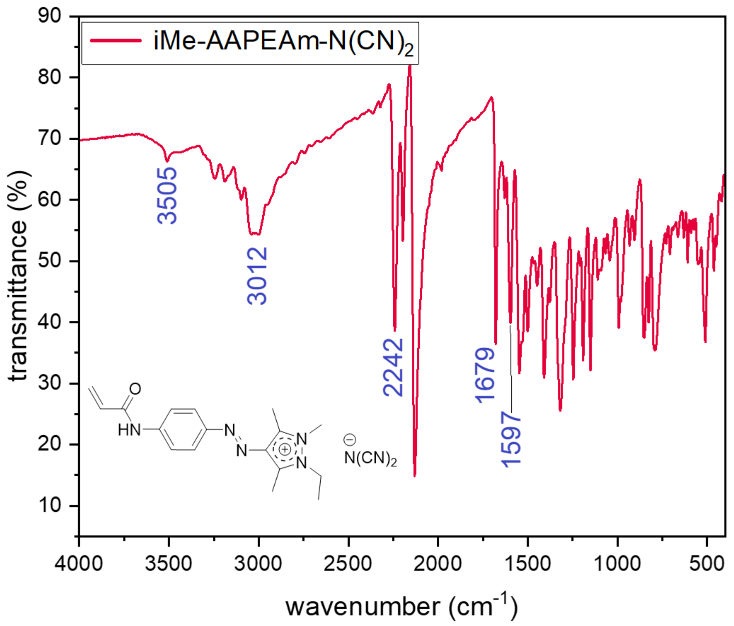

Figure S25: FT-IR spectra of monomer iMe-AAPEAm-N(CN)$_2$ (**2b**) (ATR, 298K): Blue numbers indicate wavenumbers with corresponding functional groups or bonds.

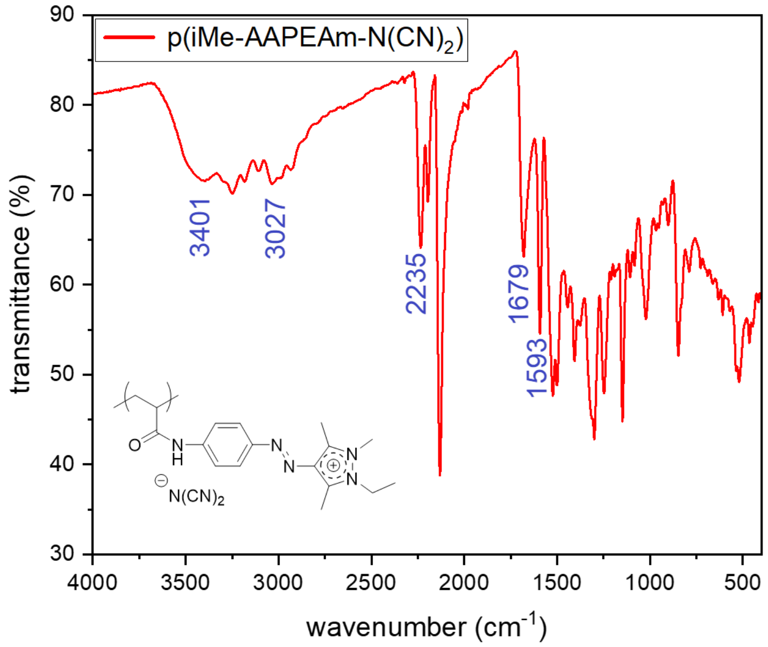

Figure S26: FT IR of polymer p(iMe-AAPEAm-N(CN)$_2$) (**P-2b**) (ATR, 298 K): Blue numbers indicate wavenumbers with corresponding functional groups or bonds.

Optical Transparency / Visual Appearance

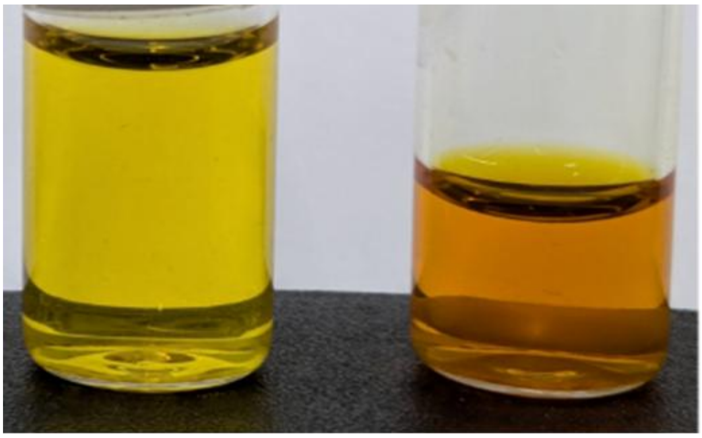

1 g·L^{-1} 5 g·L^{-1}

Figure S27: Aqueous solution of polymer p(iMe-AAPEAm-N(CN)$_2$) (**P-2b**) with a concentration of 1 g·L^{-1} (left) or 5 g·L^{-1}(right) in water at ambient temperature.

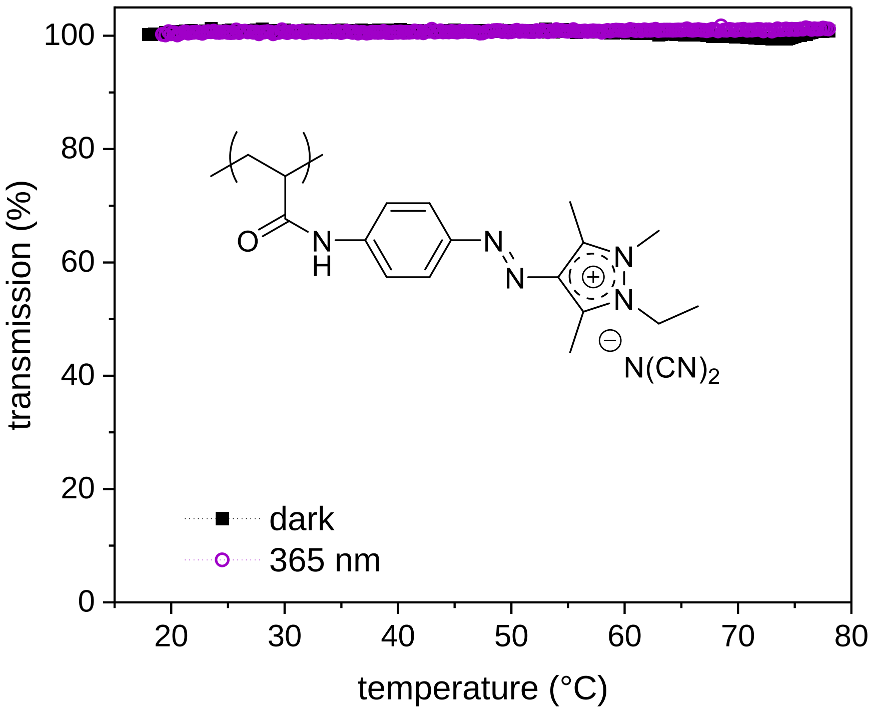

Figure S28: Turbidity study of dissolved polymer p(iMe-AAPEAm-N(CN)$_2$ (**P-2b**) (c = 1 g·L^{-1} in water) as function of the temperature and of the state of the photo-isomerization, heating rate 0.5 K min^{-1}. Black squares indicate solutions equilibrated in the dark (all in *E*-state), purple hollow circles in the photo-stationary state after 15 min of irradiation by 365 nm light, containing a Z-isomer majority. No cloud or clearing point is observed.

UV-vis Spectral Properties

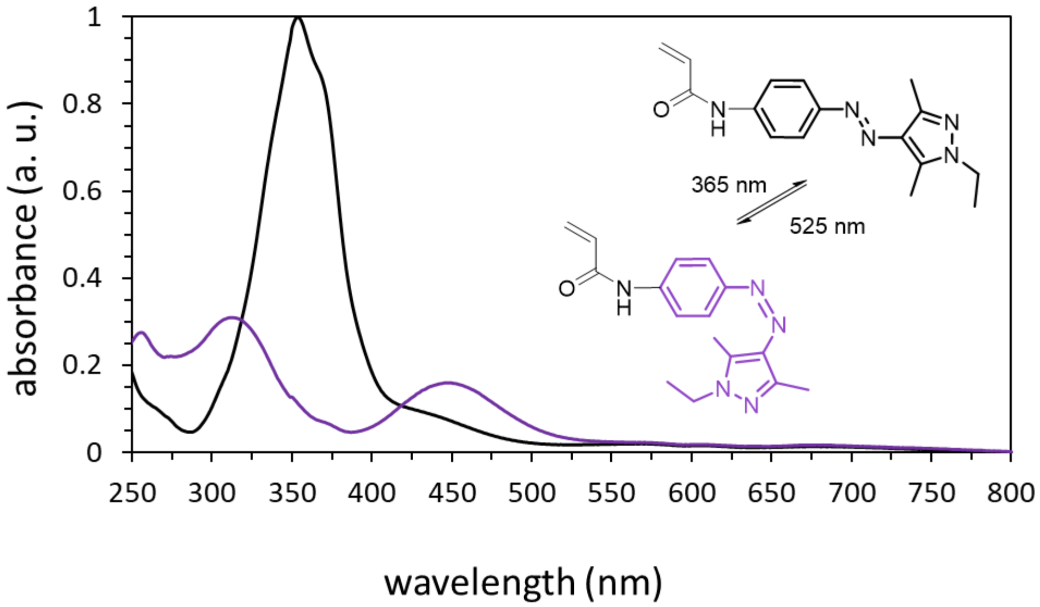

Figure S29: UV-vis spectra of non-ionic monomer AAPEAm (**1**) in methanol (0.005 g·L^{-1}) before irradiation (black) and after irradiation at 365 nm for 5 min (purple).

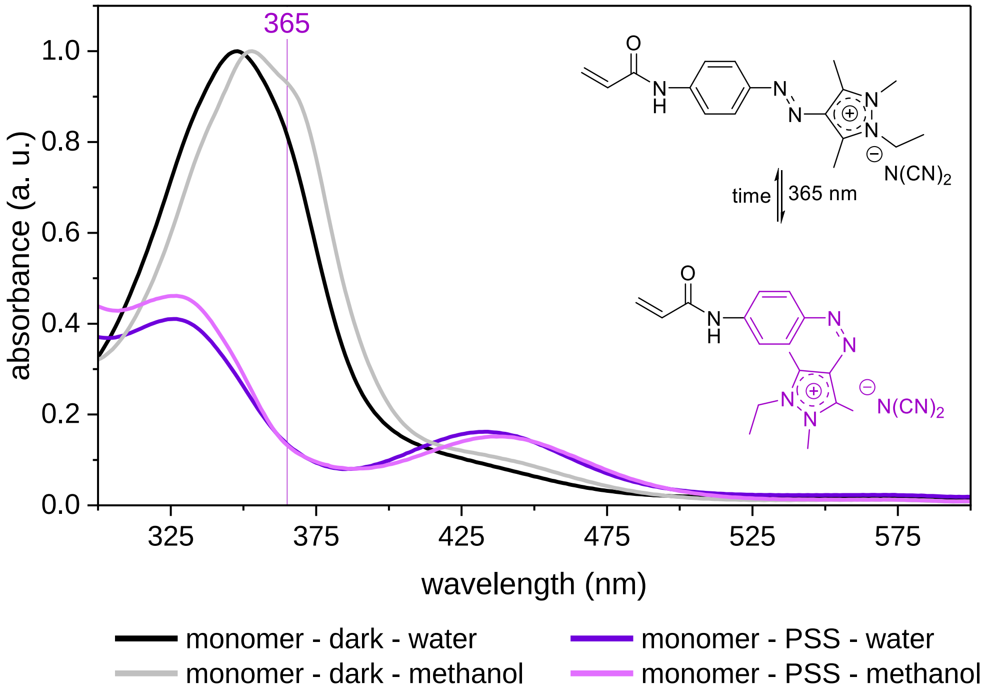

Figure S30: Comparison of the cationic monomer iMe-AAPEAm-N(CN)$_2$ (**2b**) in water (concentration = 0.004 g·L^{-1}) and in methanol (concentration = 0.06 g·L^{-1}) before (dark) and after irradiation at 365 nm for 10 min (PSS). The thin purple vertical line indicates the irradiation wavelength.

E-isomer content after irradiation

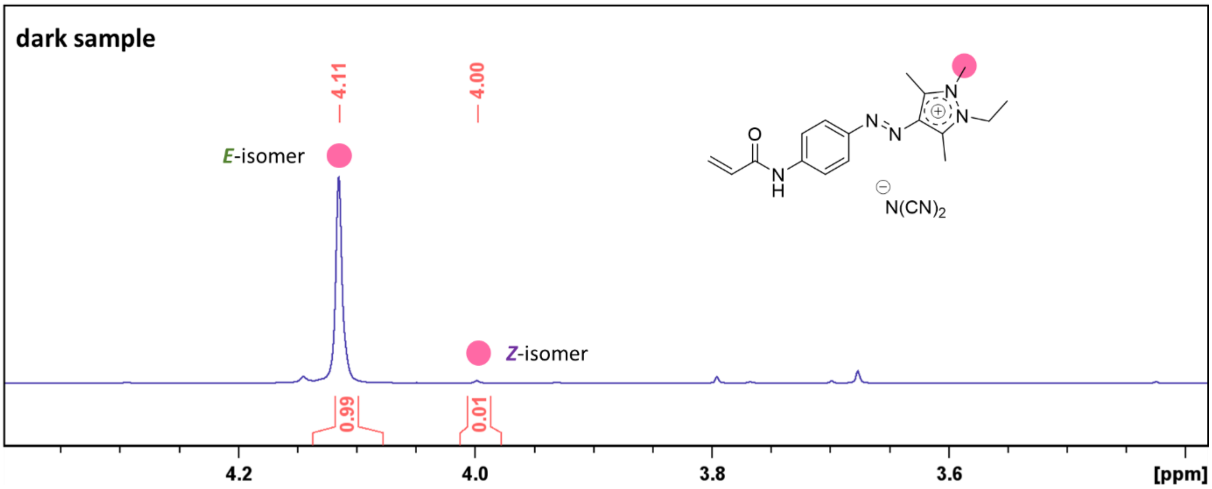

Figure S31: ^{1}H NMR of monomer iMe-AAPEAm-N(CN)$_2$ (**2b**) (400 MHz, MeOD-D$_4$, 298 K) after 1 d of annealing in the dark: The pink circles mark the protons and their corresponding signal for both isomers. The observed *E*-isomer content is 99 %.

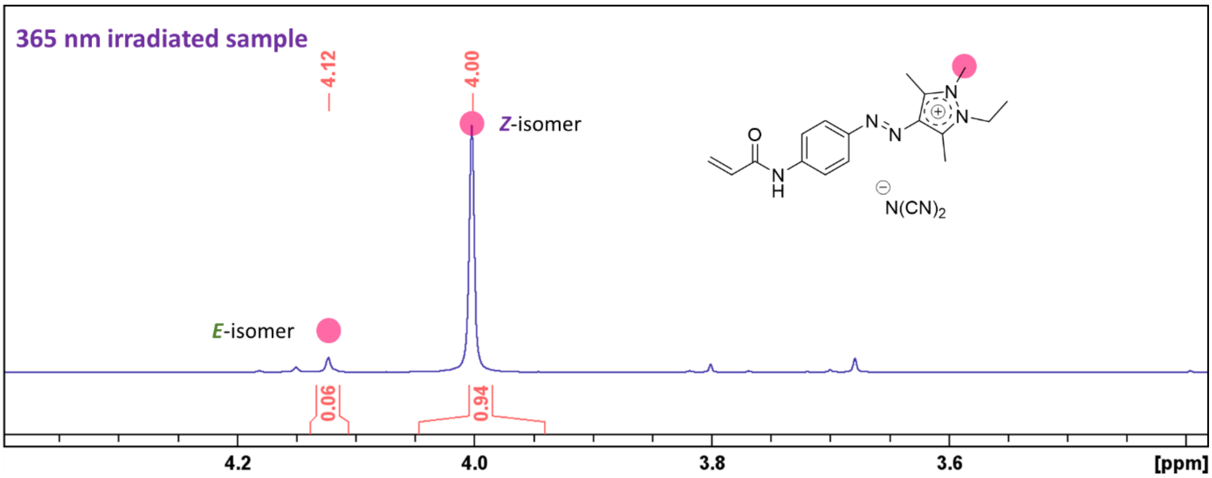

Figure S32: ^{1}H NMR of monomer iMe-AAPEAm-N(CN)$_2$ (**2b**) (400 MHz, MeOD-D$_4$, 298 K) after 15 min irradiation at 365 nm: The pink circles mark the protons and their corresponding signal for both isomers. The observed *E*-isomer content is 6 %.

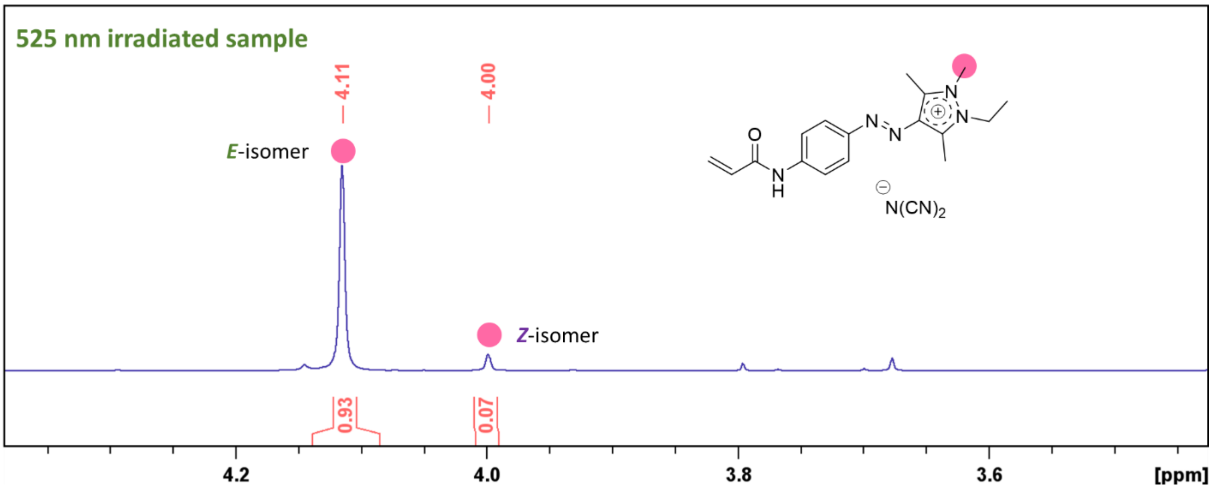

Figure S33: ^{1}H NMR of monomer iMe-AAPEAm-N(CN)$_2$ (**2b**) (400 MHz, MeOD-D$_4$, 298 K) after 15 min irradiation at 525 nm: The pink circles mark the protons and their corresponding signal for both isomers. The observed *E*-isomer content is 99 %.

Half-life time of the chromophore

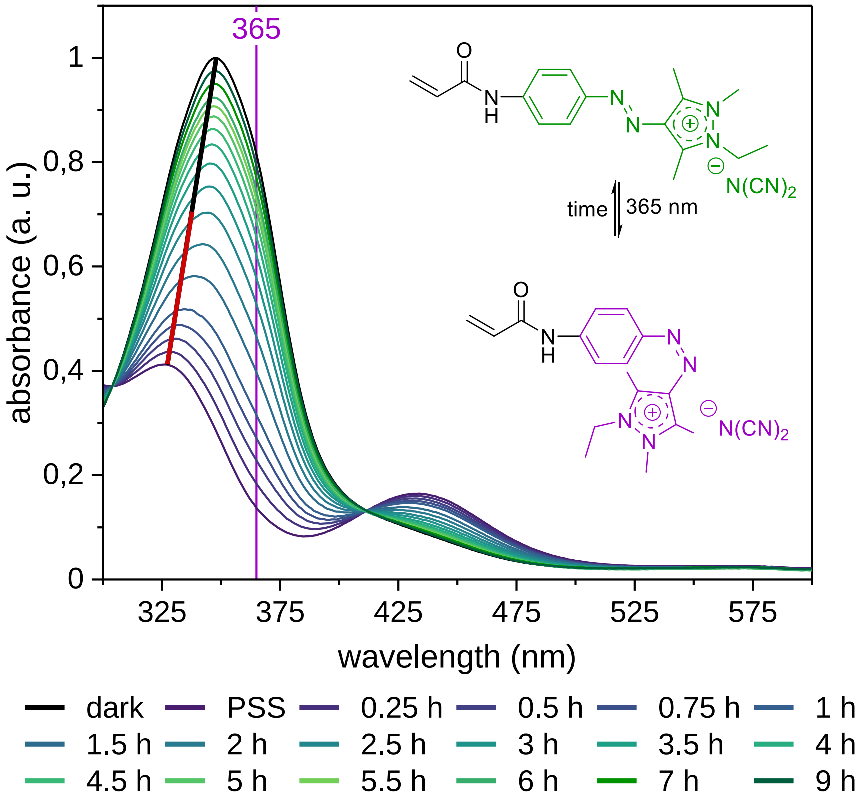

Figure S34: UV-vis-spectra showing the relaxation of the photo-stationary state of monomer iMe-AAPEAm-$N(CN)_2$ (**2b**) upon UV-irradiation in water (0.004 g·L^{-1}) over time. The thin purple vertical line indicates the position of the irradiation wavelength. The sample was measured before irradiation (dark), immediately after 5 min of UV-light irradiation (PSS), and then at fixed intervals. The black line represents the difference between the dark state and the PSS. The red-and-black line is meant as a guide to the eye for the ongoing evolution from the PSS back to the dark state, the red part representing the first half of the relaxation and ending at the halfway point, marking the estimated half-life.

Calculation of Half-life time

The decay of the photo-stationary states follows 1st order kinetics. Accordingly, the half-life time $\tau_{½}$ can be calculated from the slope of the plotted normalized ^1H NMR signals, respectively as a function of time, using equation (1).

$$\tau_{½} = \frac{\ln(2)}{slope} \tag{1}$$

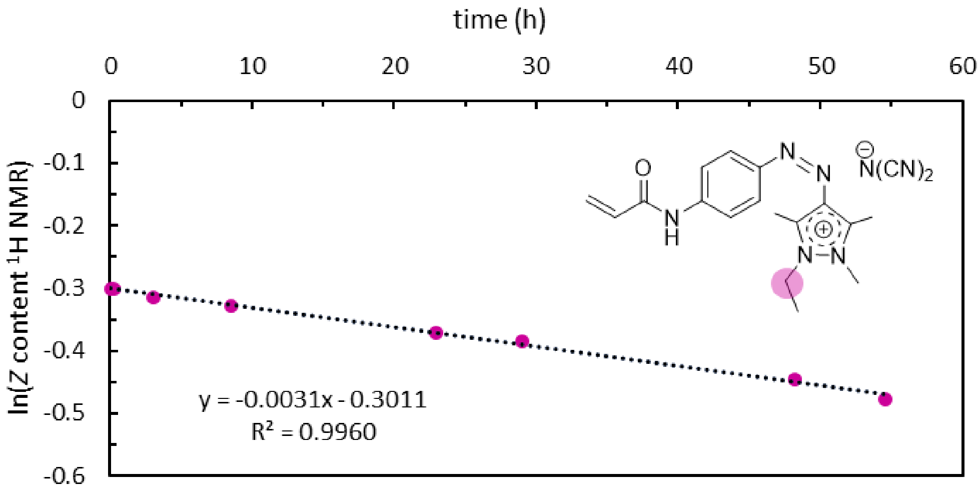

Figure S35: Linearized decay of the signal of the marked methylene group at 4.5 ppm nm for monomer iMe-AAPEAm-N(CN)$_2$ (**2b**) after irradiation with UV-light in MeOD-D$_4$ at 20°C. Using the slope of the linear regression, a half-life of 224 h (around 9 d) is calculated using equation (1).

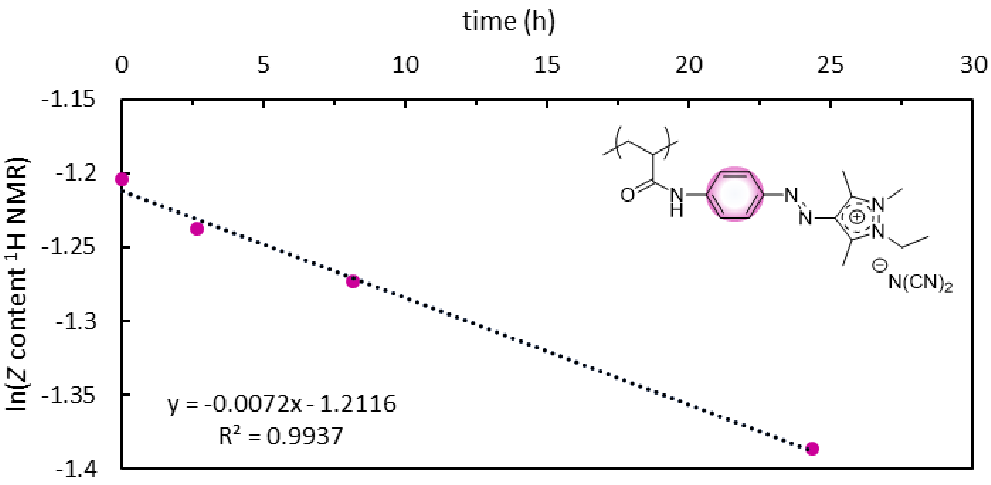

Figure S36: Linearized decay of the signal of the marked aryl group at 7.7 ppm nm for polymer p(iMe-AAPEAm-N(CN)$_2$) (**P-2b**) after irradiation with UV-light in MeOD-D$_4$ at 20 °C. Using the slope of the linear regression, a half life time of 96 h (around 4 d) is calculated using equation (1).

DLS Data

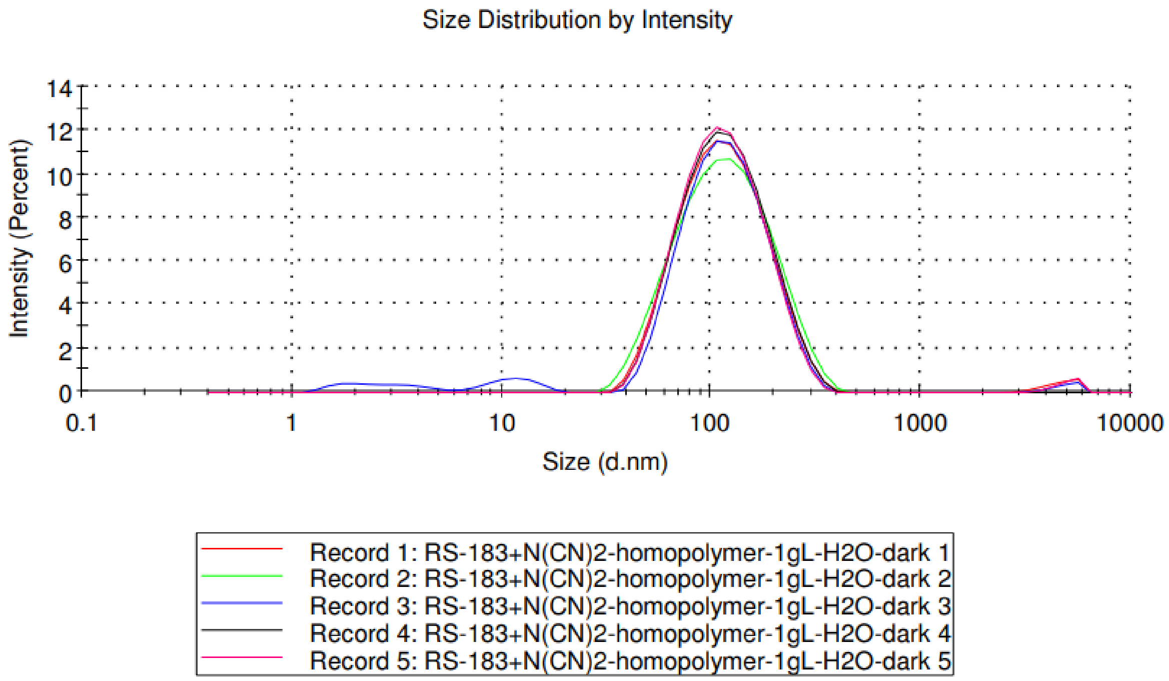

Figure S37: Particle size measurement via DLS of polymer p(iMe-AAPEAm-N(CN)$_2$) (**P-2b**) in water (1 g·L^{-1}). Before measurement, the sample was filtered through a 0.2 µm syringe filter.

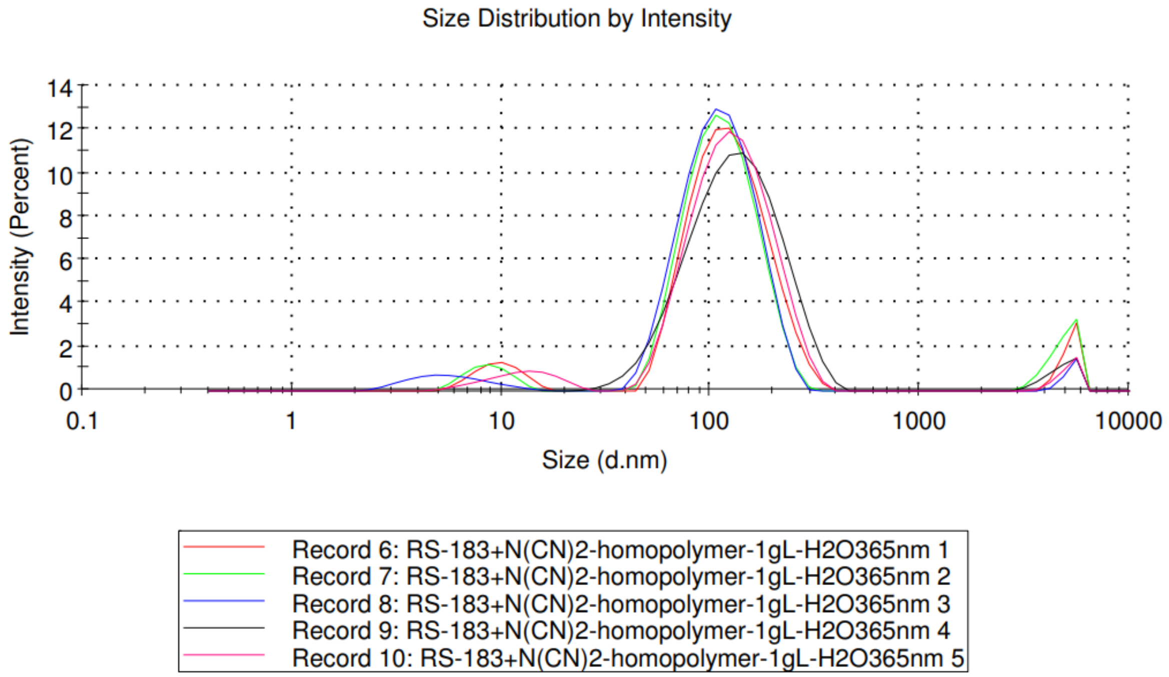

Figure S38: Particle size measurement via DLS of polymer p(iMe-AAPEAm-N(CN)$_2$) (**P-2b**) in water (1 g·L^{-1}). Before measurement, the sample was filtered through a 0.2 µm syringe filter and irradiated with UV-light (365 nm).

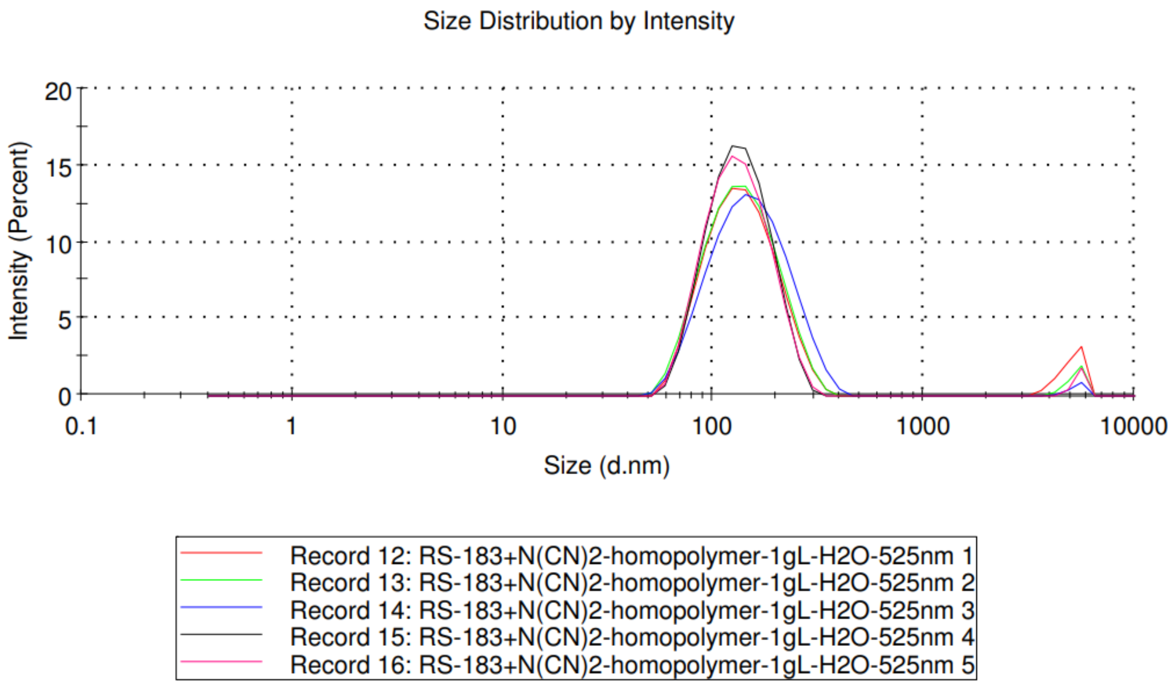

Figure S39: Particle size measurement via DLS of p(iMe-AAPEAm-N(CN)$_2$) (**P-2b**) in water (1 g·L^{-1}). Before measurement, the sample was filtered through a 0.2 µm syringe filter and irradiated with green light (525 nm).

Cryogenic scanning electron microscopy (Cryo-SEM) imaging of aqueous polymer samples

Aqueous samples of polymer p(iMe-AAPEAm-N(CN)$_2$), **P-2b**, (concentration = 5 g·L^{-1}) were plunge-frozen to investigate the polymer's structural conformation in water. The sample in its clear and turbid state was analyzed, with the exact procedure described in the experimental section. For the sample in its clear state and independent of the isomeric state of the azo dye, a band or lamellae structure was visible, uniformly spanning an entire domain and changing orientation only at grain boundaries. The measured distance between lamellae and their thickness were approximately 3 µm and 140 nm, respectively (Figure S40, top). The specimen derived from the sample in its turbid state shows no large polymer aggregates, and therefore appears as a homogeneous surface (Figure S40, bottom).

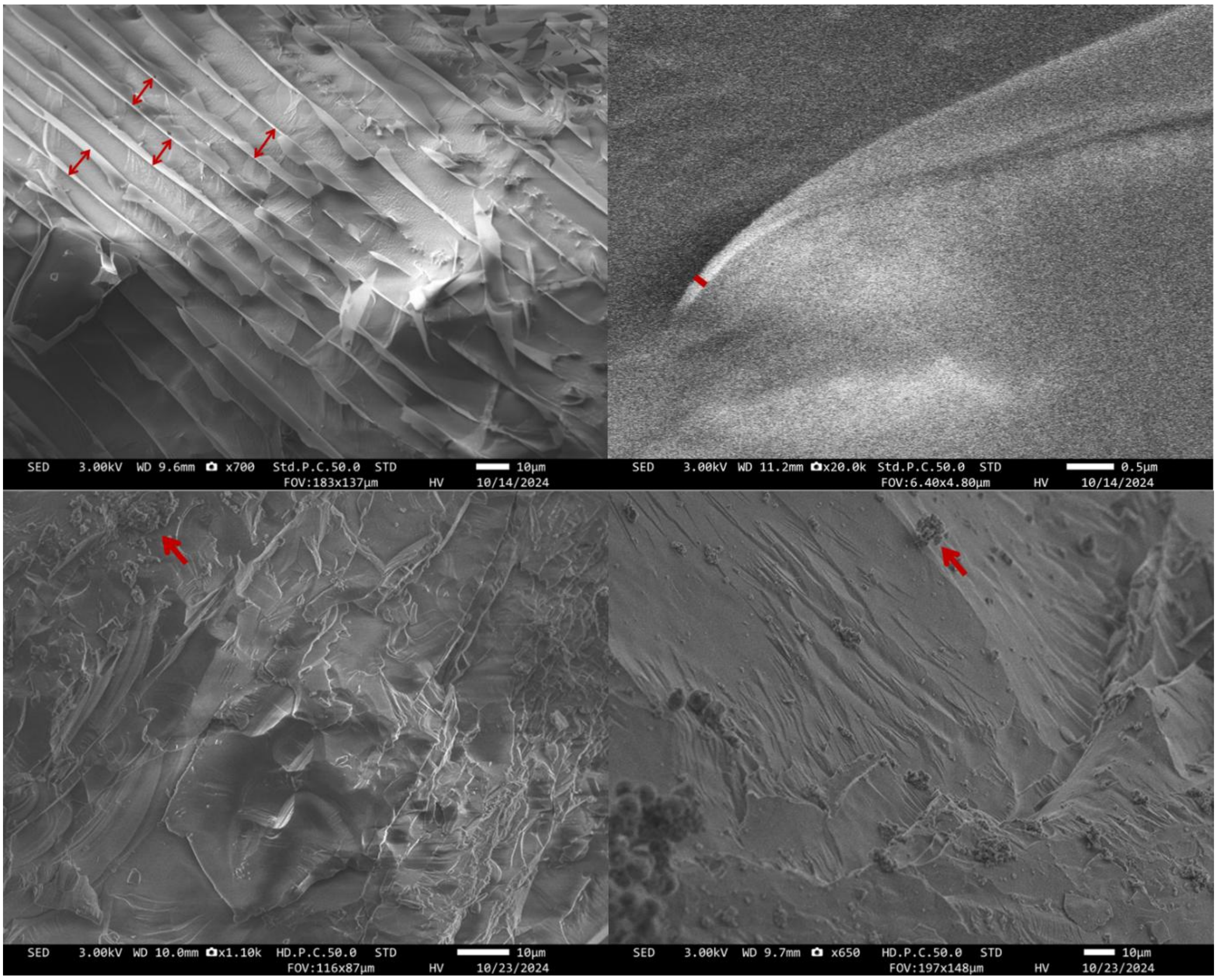

Figure S40: Top: Cryo-SEM image of plunge-frozen polymer p(iMe-AAPEAm-N(CN)$_2$) (**P-2b**) in water (clear sample, concentration = 5 g·L^{-1}). A uniform band structure is visible. Bottom: Cryo-SEM image of cracked orb of **P-2b** specimen in water (turbid sample, concentration = 5 g·L^{-1}). Red arrows indicate residual water on the orb surface. No lamellae structure is visible.

To distinguish ice regions from polymer regions, energy-dispersive spectroscopy (EDS) was performed on a specific area of the specimen with the lamella structure, derived from the clear sample focusing on carbon, nitrogen, and oxygen. While carbon and nitrogen were present in an approximately 2:1 ratio in the polymer, oxygen was present in a much lower ratio (19:1 relative to carbon) in the polymer but is abundant in the ice. The atomic count map confirms the observed lamellar structure, showing that the elements carbon and nitrogen are concentrated in the lamella regions, as shown in Figure S41. For the specimen without the lamella structure, derived from the turbid sample, no regions with increased carbon and nitrogen concentration, *i.e.*, increased polymer concentration, are observed.

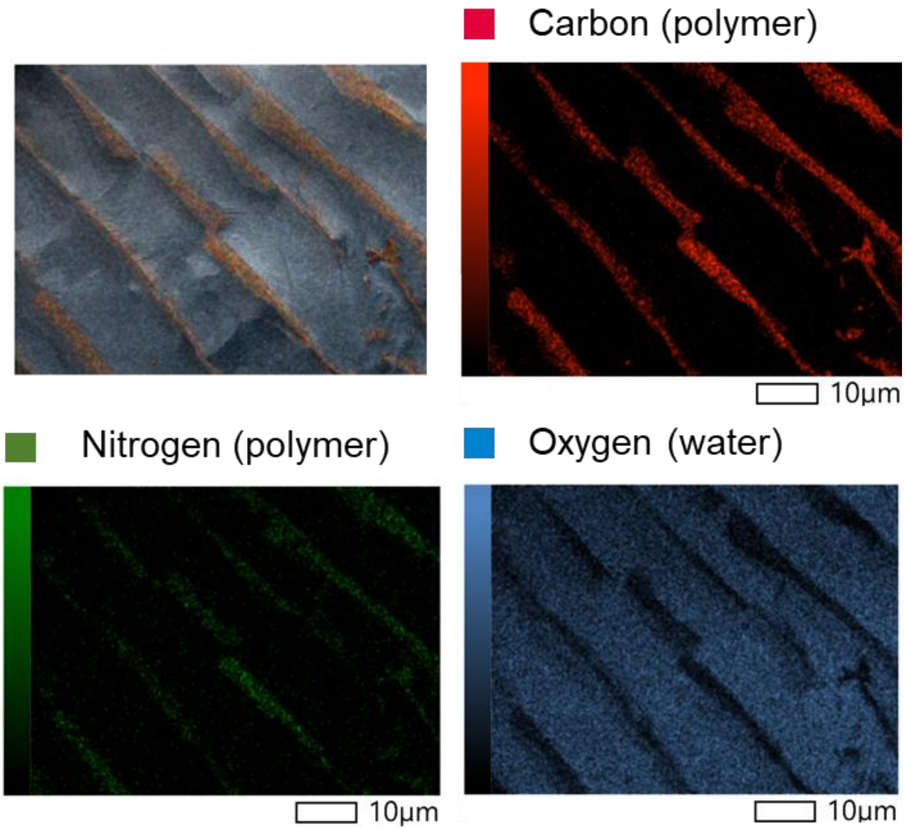

Figure S41: Atom count map of polymer p(iMe-AAPEAm-N(CN)$_2$) (**P-2b**) in water (clear sample, polymer concentration = 5 g·L^{-1}). Carbon (red) and nitrogen (green) from the polymer are concentrated in the lamella regions, while oxygen (blue) is dominant in the ice regions.

It is currently not clear, whether this structure forms during the rapid cooling process of plunge-freezing, or is already present in the clear solution. Given the large size of the lamellae, extending several hundred micrometers in length and approximately 140 nm in thickness, the latter is however unlikely, as such structures in the clear solution would be expected to scatter visible light. UV-light irradiation of the clear sample did not alter the overall structure. Large domains, each several hundred micrometers in size, still dominate the specimen's structure.

References

(1) Gaur, A. K.; Gupta, D.; Mahadevan, A.; Kumar, P.; Kumar, H.; Nampoothiry, D. N.; Kaur, N.; Thakur, S. K.; Singh, S.; Slanina, T.; Venkataramani, S. Bistable Aryl Azopyrazolium Ionic Photoswitches in Water. *J. Am. Chem. Soc.* **2023**, *145* (19), 10584–10594. DOI: 10.1021/jacs.2c13733.

(2) Liu, W.; Lin, Q.; Li, Y.; Chen, P.; Fang, T.; Zhang, R.; Pang, S. Solid-state Reaction of Azolium Hydrohalogen Salts with Silver Dicyanamide - Unexpected Formation of Cyanoguanidine-azoles, Reaction Mechanism and Their Hypergolic Properties. *Sci. Rep.* **2015**, *5*, 10915. DOI: 10.1038/srep10915.
